# Chromatin-associated microprocessor assembly is regulated by the U1 snRNP auxiliary protein PRP40

**DOI:** 10.1093/plcell/koac278

**Published:** 2022-09-10

**Authors:** Agata Stepien, Jakub Dolata, Tomasz Gulanicz, Dawid Bielewicz, Mateusz Bajczyk, Dariusz J Smolinski, Zofia Szweykowska-Kulinska, Artur Jarmolowski

**Affiliations:** Department of Gene Expression, Faculty of Biology, Institute of Molecular Biology and Biotechnology, Adam Mickiewicz University, Poznan 61-614, Poland; Department of Gene Expression, Faculty of Biology, Institute of Molecular Biology and Biotechnology, Adam Mickiewicz University, Poznan 61-614, Poland; Department of Gene Expression, Faculty of Biology, Institute of Molecular Biology and Biotechnology, Adam Mickiewicz University, Poznan 61-614, Poland; Department of Gene Expression, Faculty of Biology, Institute of Molecular Biology and Biotechnology, Adam Mickiewicz University, Poznan 61-614, Poland; Department of Gene Expression, Faculty of Biology, Institute of Molecular Biology and Biotechnology, Adam Mickiewicz University, Poznan 61-614, Poland; Department of Cellular and Molecular Biology, Nicolaus Copernicus University, Torun 87-100, Poland; Centre for Modern Interdisciplinary Technologies, Nicolaus Copernicus University, Torun 87-100, Poland; Department of Gene Expression, Faculty of Biology, Institute of Molecular Biology and Biotechnology, Adam Mickiewicz University, Poznan 61-614, Poland; Department of Gene Expression, Faculty of Biology, Institute of Molecular Biology and Biotechnology, Adam Mickiewicz University, Poznan 61-614, Poland

## Abstract

In plants, microRNA (miRNA) biogenesis involves cotranscriptional processing of RNA polymerase II (RNAPII)-generated primary transcripts by a multi-protein complex termed the microprocessor. Here, we report that Arabidopsis (*Arabidopsis thaliana*) PRE-MRNA PROCESSING PROTEIN 40 (PRP40), the U1 snRNP auxiliary protein, positively regulates the recruitment of SERRATE, a core component of the plant microprocessor, to miRNA genes. The association of DICER-LIKE1 (DCL1), the microprocessor endoribonuclease, with chromatin was altered in *prp40ab* mutant plants. Impaired cotranscriptional microprocessor assembly was accompanied by RNAPII accumulation at miRNA genes and retention of miRNA precursors at their transcription sites in the *prp40ab* mutant plants. We show that cotranscriptional microprocessor assembly, regulated by AtPRP40, positively affects RNAPII transcription of miRNA genes and is important to reach the correct levels of produced miRNAs.

## Teaser

PRP40 contributes to the cotranscriptional recruitment and activity of microprocessor components.

IN A NUTSHELL **Background:** MicroRNAs (miRNAs) are single-stranded, usually 21-nt-long, RNAs that regulate basic developmental processes as well as plant responses to fluctuations in environmental conditions. Plant miRNA genes (*MIR*s) are transcribed by RNA polymerase II (RNAPII) to generate long precursors that are processed into mature miRNAs by the miRNA biogenesis complex known as the microprocessor.

**Question:** We have previously demonstrated that processing of plant miRNA precursors takes place already during their synthesis by RNAPII. However, the mechanism of regulation of microprocessor assembly on primary transcripts of *MIR*s was unknown.

**Findings:** In these studies, we show that the auxiliary U1 snRNP protein AtPRP40 interacts directly with the C-terminal domain of RNAPII as well as one of the microprocessor components, and coordinates the binding of microprocessor elements to miRNA primary precursors at the sites of their synthesis. We show that the impaired cotranscriptional microprocessor assembly that is observed in the absence of AtPRP40 is accompanied by RNAPII accumulation at miRNA genes and retention of miRNA precursors at their transcription sites. Our data suggest that cotranscriptional microprocessor assembly, regulated by AtPRP40, positively affects RNAPII transcription of miRNA genes.

**Next steps:** The obtained results raise interesting questions about a role of AtPRP40 in coordinating transcription and other processing events. Further studies will explore the influence of AtPRP40 on processes other than miRNA biogenesis.

## Introduction

MicroRNAs (miRNAs) are single-stranded, usually 21-nt-long, RNAs that regulate basic developmental processes as well as plant responses to constant fluctuations in environmental conditions (Kruszka et al., 2012; Barciszewska-Pacak et al., 2015; Borges and Martienssen, 2015; Yu et al., 2017). Therefore, miRNA production has to be tightly controlled at multiple levels (Stepien et al., 2017; Dolata et al., 2018). Plant miRNA genes (*MIR*s) are transcribed by RNA polymerase II (RNAPII) to generate precursors that are subsequently cleaved to miRNA/miRNA* duplexes. In plants, both steps of miRNA biogenesis are carried out in the nucleus by the RNase III-type ribonuclease DICER-LIKE1 (DCL1) (Kurihara and Watanabe, 2004). Depending on the miRNA primary precursor (pri-miRNA) structure, DCL1 generates the first cut near the base of the hairpin structure (base-to-loop [BTL] processing) or starts from the hairpin loop (loop-to-base processing, LTB) (Zhu et al., 2013; Moro et al., 2018). DCL1 is assisted by SERRATE (SE) and HYPONASTIC LEAVES1 (HYL1) for accurate and efficient activity (Dong et al., 2008; Laubinger et al., 2008; Szarzynska et al., 2009). These three proteins, DCL1, SE, and HYL1, form the core of the plant complex that processes miRNAs, also known as the microprocessor.

Similar to that of animals, plant pri-miRNA cleavage was recently reported to occur cotranscriptionally (Morlando et al., 2008; Gonzalo et al., 2022). The core components of the microprocessor are known to be associated with chromatin. The association of DCL1 with *MIR*s is mediated by the Mediator and Elongator complexes (Fang et al., 2015; Cambiagno et al., 2021). PP4R3A, the regulatory subunit of PROTEIN PHOSPHATASE4 (PP4), was shown to promote HYL1 association with *MIR* promoters (Wang et al., 2019), and THREE PRIME REPAIR EXONUCLEASE 2 (TREX-2) contributes to the DCL1 and HYL1 recruitment to *MIRs* (Zhang et al., 2020). SE was also found to bind and regulate the transcription of intronless genes (Speth et al., 2018). However, the cotranscriptional mechanism of plant microprocessor assembly and its interplay with RNAPII activity is still unclear and requires investigation.

We previously showed that the SE and U1 snRNP proteins are responsible for the communication between the microprocessor and the splicing machinery (Knop et al., 2017). Among the U1 snRNP partners of SE, PRE-MRNA PROCESSING PROTEIN 40 (PRP40) is particularly interesting. Since U1 snRNP is recruited cotranscriptionally to intron-containing genes in yeast shortly downstream from the 5′ splice site, U1 snRNP elements have been considered potential players in the well-established crosstalk between the splicing and transcription machinery (Greenleaf, 1993; McCracken et al., 1997; Kotovic et al., 2003; Lacadie and Rosbash, 2005; Görnemann et al., 2005; Tardiff and Rosbash, 2006; Braunschweig et al., 2013). The reported direct interaction of yeast PRP40p with the RNAPII C-terminal domain (CTD) (Morris and Greenleaf, 2000) indicates that this protein is a good candidate for such communication. Plant and animal PRP40 homologs can also interact with CTD (Suñé et al., 1997; Carty et al., 2000; Kang et al., 2009). However, the published data suggest that the main role of PRP40 is stabilization of U1 snRNP by the interactions of PRP40 with other U1 components and modulation of alternative splicing (Kao and Siliciano, 1996; Morris and Greenleaf, 2000; Ester and Uetz, 2008; Görnemann et al., 2011; Becerra et al., 2015; Choudhary et al., 2021), and its possible function in the cotranscriptional recruitment of different macromolecular complexes to RNAPII is still an open question.

We previously suggested that PRP40 may be involved in cotranscriptional microprocessor assembly due to the direct interaction between AtPRP40 and SE (Knop et al., 2017). Here, we found that the levels of almost half of the polyadenylated pri-miRNAs were affected in *prp40ab* (the majority of which were downregulated). However, in parallel, we observed increased levels of chromatin-associated nascent *MIR* transcripts. Interestingly, mature miRNAs, on average, showed only slightly increased levels in *prp40ab*. We demonstrate that the higher accumulation of RNAPII along pre-miRNA genes agrees with the altered recruitment of microprocessor components, SE and DCL1, to *MIR*s. Our results show that in plants, PRP40 is involved in the coordination of the microprocessor assembly on pri-miRNAs while they are synthesized by RNAPII.

## Results

### AtPRP40 is important for *Arabidopsis thaliana* development

The Arabidopsis (*A. thaliana*) genome has three *PRP40* genes (Wang and Brendel, 2004) that show differences in expression levels in various tissues and developmental stages (Supplemental Figure S1). *AtPRP40A* showed the highest expression, whereas *AtPRP40C* was barely expressed in all tested samples. In our previous studies, we identified AtPRP40a and AtPRP40b as proteins involved in the interplay between miRNA production and the splicing of miRNA precursors (Knop et al., 2017). Interestingly, when we assessed the colocalization of AtPRP40b and a core component of U1 snRNP U1 snRNA, we found low colocalization coefficients (Supplemental Figure S2). This finding suggests an additional activity of AtPRP40 beyond the U1 snRNP complex.

Single mutants of each *AtPRP40* did not differ from wild-type (WT) plants (Supplemental Figure S3, a and b). However, the double *prp40a prp40b* (hereafter *prp40ab*) mutant showed delayed growth compared with WT plants (Supplemental Figure S3, c and d). Interestingly, the *AtPRP40A* mRNA level in the single *prp40b* mutant was elevated compared with that in the WT plants, and similarly, *AtPRP40B* mRNA expression was higher in the *prp40a* mutant than in the WT plants (Supplemental Figure S4a). This result was also confirmed at the protein level using antibodies recognizing AtPRP40b (Supplemental Figure S4b). Notably, the level of *AtPRP40C* mRNA was not different in *prp40a*, *prp40b*, or *prp40ab* compared with the level in WT plants (Supplemental Figure S4a). Since we were not able to obtain homozygotes of the triple *prp40a prp40b prp40c* mutant (most likely due to a relatively short distance between the *AtPRP40B* and *AtPRP40C* genes, approximately 54 kb), for further analyses we used the double *prp40ab* mutant.

Interestingly, we found that the crosstalk between SE and AtPRP40a and AtPRP40b is also crucial for plant development, as the mutation of *SE* and inactivation of *AtPRP40A* and *AtPRP40B* led to embryo lethality (Supplemental Figure S5). Notably, we did not observe this phenomenon after crossing *prp40ab* with *hyl1-2*, a mutant of another core microprocessor component, HYL1. Because *hyl1-2* plants have ovule defects (Wei et al., 2020), we also compared the embryo lethality observed in the *hyl1-2* mutant with that of the *prp40ab* × *hyl1-2* cross. Interestingly, we found partial restoration of the WT phenotype in the *prp40ab × hyl1-2* mutant compared with the single *hyl1-2* mutant plants (Supplemental Figure S5).

### AtPRP40 mediates the association of SE with RNAPII and *MIR*s

**Figure 1:**
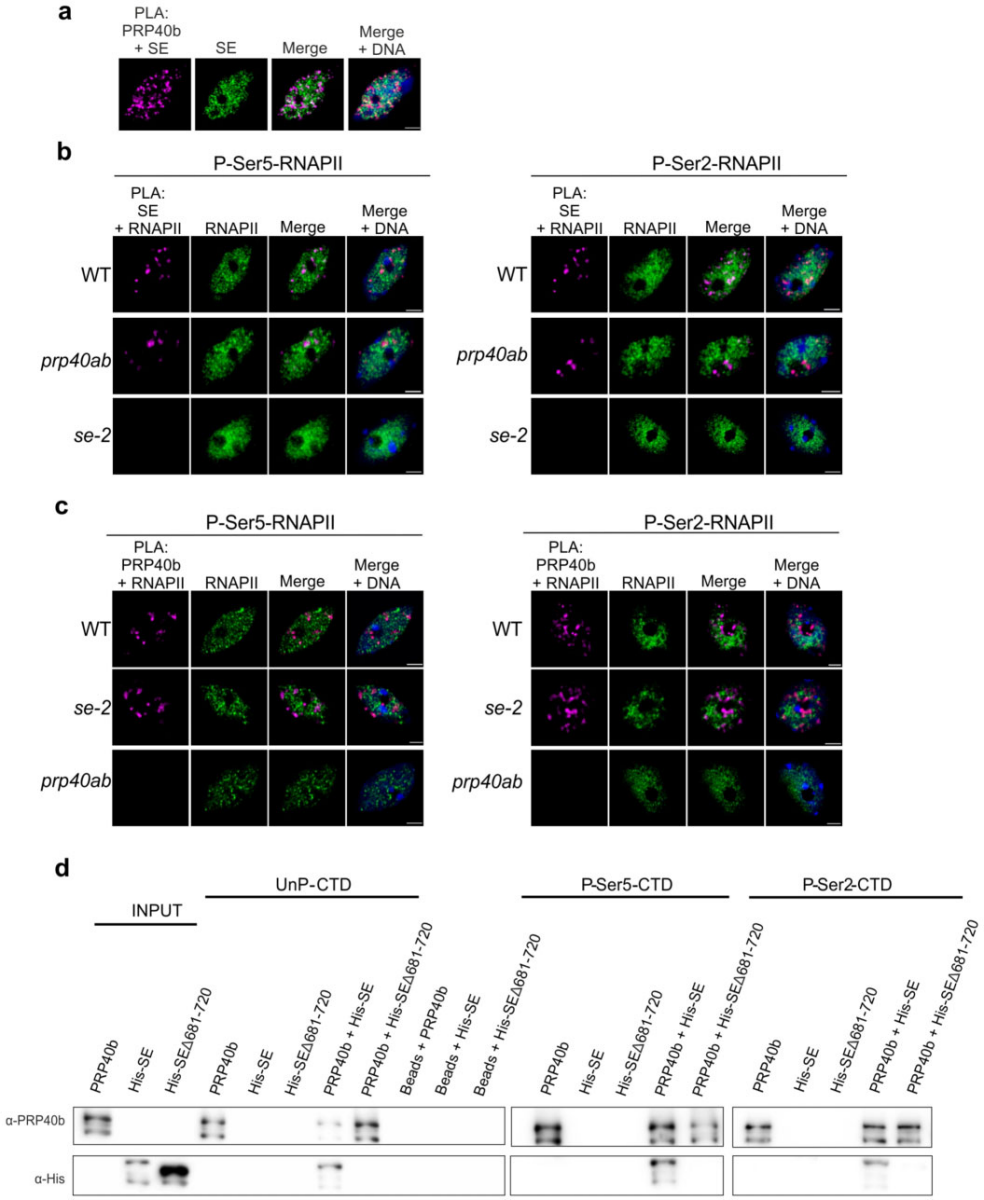
AtPRP40 regulates the association of SE with RNAPII. A, Close proximity of AtPRP40b and SE in the cell nucleus (first image, magenta signals) analyzed by PLA in view of SE nuclear localization (second image, green signals). DNA was stained with Hoechst (blue). Scale bar = 2.5 µm. B, Close proximity of SE and RNAPII phosphorylated at CTD Ser5 or Ser2 in WT, *prp40ab*, and *se-2* plants (first columns, magenta signals) in view of P-Ser5-RNAPII or P-Ser2-RNAPII nuclear localization (second columns, green signals) detected by PLA. DNA was stained with Hoechst (blue). Scale bar = 2.5 µm. C, Close proximity of AtPRP40b and RNAPII phosphorylated at CTD Ser5 or Ser2 in WT, *se-2*, and *prp40ab* plants (first columns, magenta signals) in view of P-Ser5-RNAPII or P-Ser2-RNAPII nuclear localization (second columns, green signals) detected by PLA. DNA was stained with Hoechst (blue). Scale bar = 2.5 µm. D, In vitro pull-down assays using recombinant AtPRP40b and SE proteins (both full length and the shortened C-terminus variant Δ681–720 were used) and biotinylated CTD peptides in unphosphorylated (UnP) or phosphorylated Ser5 (P-Ser5-CTD) or Ser2 (P-Ser2-CTD) forms. The recombinant proteins were incubated with CTD peptides immobilized on beads. The obtained complexes were washed, eluted, and analyzed by immunoblot. Input represents 1/10 of the protein sample.

Since SE was shown to associate with RNAPII (Speth et al., 2018), we investigated whether this contact is regulated by AtPRP40. We performed colocalization analyses and utilized the proximity-ligation assay (PLA). We observed clear PLA foci that indicate close proximity of AtPRP40b and SE in the Arabidopsis cell nucleus (Figure 1A). This result was confirmed by overlapping of SE and AtPRP40b localization signals in the immunolocalization experiment (Supplemental Figure S6). Next, by utilizing PLA and immunolocalization, we tested the close proximity and colocalization of SE with RNAPII phosphorylated at both Ser5 (P-Ser5-RNAPII) and Ser2 (P-Ser2-RNAPII) (Figure 1B and Supplemental Figure S7). The colocalization coefficients calculated for SE and both phosphorylated forms of RNAPII were significantly lower in the *prp40ab* mutant plants than in WT plants (Supplemental Figure S7b), and the PLA signal numbers also decreased in *prp40ab* mutant plants compared with in WT plants (Figure 1B), indicating that AtPRP40 mediates the association of SE with RNAPII.

In contrast, we did not observe any change, either in the colocalization coefficients or the PLA signals, while analyzing the association of AtPRP40b and RNAPII in *se-2* mutant plants (Supplemental Figure S8 and Figure 1C). These results indicate that the colocalization and close proximity of AtPRP40b with RNAPII do not depend on SE.

Next, we performed an in vitro pull-down assay in which peptides containing four repeats of RNAPII CTD heptapeptide in their phosphorylated and unphosphorylated forms were used, as well as recombinant AtPRP40b and SE proteins overexpressed in *Escherichia coli.* Using this pull-down, we showed that SE forms a complex with the CTD of both the unphosphorylated and phosphorylated forms of RNAPII only in the presence of AtPRP40b (Figure 1D). We did not observe any direct interaction between SE and CTD without AtPRP40b. In this experiment, we also generated the SE protein lacking 40 C-terminal amino acids (SEΔ681-720) that corresponds to the SE variant expressed in the *se-2* mutant. We previously showed that this truncated SE protein cannot bind to AtPRP40b (Knop et al., 2017); thus, we used this SE variant as a negative control. Indeed, we observed SE-AtPRP40b-CTD complex formation only in the presence of the full-length SE but not when the SEΔ681-720 shortened variant of SE was used (Figure 1D).

**Figure 2:**
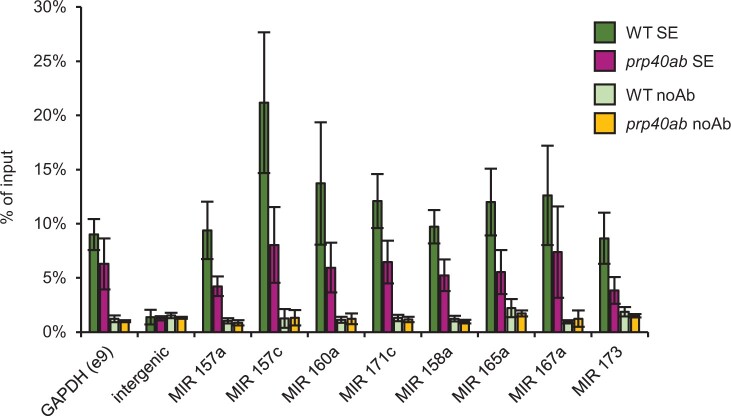
AtPRP40 is required for the proper accumulation of SE on *MIR* genes. ChIP-qPCR analyses of SE accumulation on randomly selected miRNA genes in WT and *prp40ab* mutant plants. Data represent means ± sd (*n* = 3). Primers amplifying pri-miRNA/pre-miRNA coding regions were used.

Furthermore, we performed a chromatin immunoprecipitation (ChIP) experiment to determine SE recruitment to miRNA genes in *prp40ab* mutant plants. We found lower SE accumulation on all *MIR*s tested in *prp40ab* than in the WT plants (Figure 2 and Supplemental Figure S9).

Our data indicate that AtPRP40 regulates the recruitment of SE to RNAPII and *MIR*s and may be involved in the regulation of miRNA biogenesis.

### AtPRP40 is involved in the transcription of pri-miRNAs

**Figure 3:**
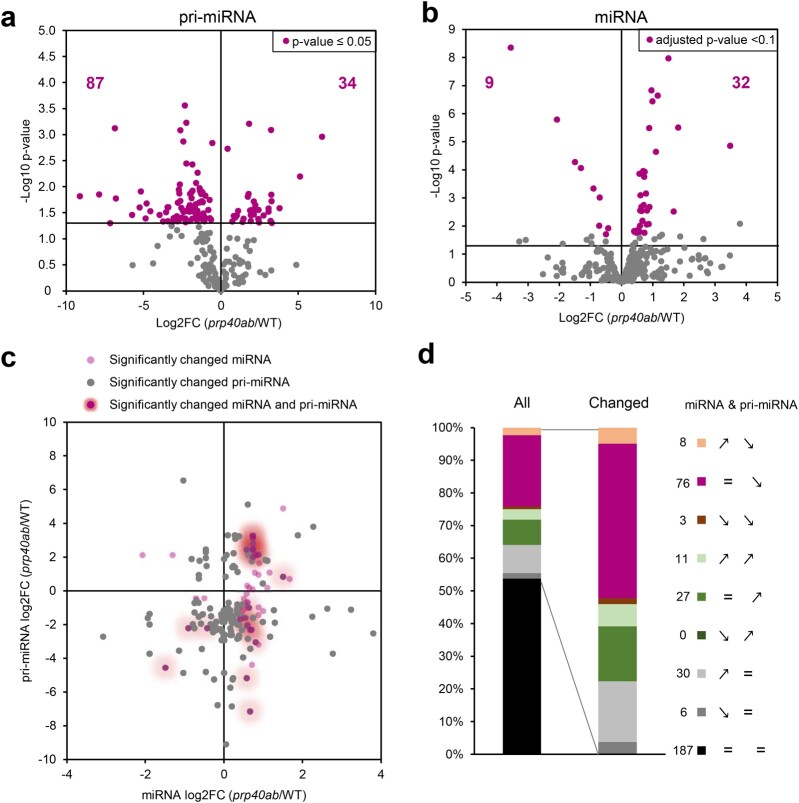
AtPRP40 affects the early steps of miRNA biogenesis. A, Volcano plot showing changes in the levels of polyadenylated *MIR* transcripts in *prp40ab* plants compared with in WT plants (RT-qPCR, *n* = 3). B, Volcano plot showing changes in the levels of miRNAs in *prp40ab* plants compared with in WT plants (small RNA sequencing, *n* = 3). C, Scatter plot showing the relationship between the levels of polyadenylated *MIR* transcripts and corresponding miRNAs. D, Expression patterns in *prp40ab* plants for polyadenylated *MIR* transcripts paired with the corresponding miRNAs. Numbers on the legend correspond to miRNA and pri-miRNA pairs from each group. Signs on the legend indicate the expression pattern in *prp40ab* plants compared with in WT plants for miRNA and pri-miRNA, respectively (↗ increased level, ↘ decreased level, and = not changed).

To test the role of AtPRP40 in miRNA production, we applied our high-throughput RT-qPCR platform, mirEX 2.0, which allowed us to analyze the levels of 297 Arabidopsis pri-miRNAs (Bielewicz et al., 2012; Zielezinski et al., 2015). The levels of 46% of the polyadenylated pri-miRNAs were significantly changed in *prp40ab* (121 out of 261 pri-miRNAs which we were able to detect) compared with WT (Figure 3A). The majority (71%) of affected precursors showed downregulated expression (87 out of 121 pri-miRNA significantly affected). The affected pri-miRNAs belong to either low- or high-expression pri-miRNAs; thus, AtPRP40 protein activity does not depend on the pri-miRNA expression level (Supplemental Figure S10a). Moreover, when we analyzed the pri-miRNA levels in *prp40ab* with regard to the presence or absence of an intron (or introns) in the *MIR* gene, we did not find any clear patterns (Supplemental Figure S10b). Further, using a decay assay with cordicepin, we excluded the possibility that the decreased level of miRNA precursor in *prp40ab* was due to changes in the stability of the poly(A)-tailed pri-miRNAs (Supplemental Figure S11). The half-lives of several pri-miRNAs tested were not changed in the *prp40ab* mutant in comparison to in WT plants. The reduced level of polyadenylated pri-miRNAs was specific for double *prp40ab* mutant plants, since we did not observe a similar effect in single *prp40a*, *prp40b*, or *prp40c* mutants (Supplemental Figure S12).

To determine how this decreased level of polyadenylated pri-miRNAs in *prp40ab* mutant plants affects mature miRNAs, we performed small RNA sequencing and compared the miRNA levels of the WT and *prp40ab* plants. Surprisingly, the levels of most of the mature miRNAs were not changed or were even slightly upregulated in the *prp40ab* mutants compared with in WT plants (Figure 3B). The significantly changed miRNAs were those present in the highest amounts (Supplemental Figure S10c). Interestingly, the miRNAs showing no change or upregulated levels correspond mainly to the poly(A) pri-miRNAs that showed lower levels in *prp40ab* (Figure 3, C and D). Furthermore, we investigated whether precision of miRNA production is impaired in *prp40ab* plants, and we did not observe any bias in the *prp40ab* mutants compared with in the WT plants (Supplemental Figure S10d). To elucidate this unexpected phenomenon, we randomly selected a group of *MIR*s (including polyadenylated *MIR* transcripts with upregulated, downregulated, and unaffected levels) and tested their expression levels using cDNA templates synthesized with random hexamer primers instead of oligo d(T), which was used to monitor the levels of poly(A)-tailed pri-miRNAs. Surprisingly, with this approach, the *MIR* transcript levels were not changed in *prp40ab* plants compared with in WT plants (Supplemental Figure S10e).

We wanted to exclude the possibility that the effect on poly(A)-tailed pri-miRNA levels was due to the change in the amount of DCL1, a major component of the plant microprocessor. To this end, we performed an immunoblot analysis in WT and the *prp40ab* plants. The levels of DCL1 and SE, a second key subunit of the microprocessor, were not altered in *prp40ab* plants compared with in WT plants (Supplemental Figure S13). However, we observed an increased level of the double-stranded RNA-binding protein HYL1 in *prp40ab* compared with WT plants.

Thus, these results suggest that the lack of Arabidopsis PRP40a and b affects the accumulation of polyadenylated pri-miRNAs, but the final effect on the production of mature miRNA in *prp40ab* mutants is rather minor.

### AtPRP40 affects RNAPII and DCL1 occupancy on *MIR* genes

**Figure 4:**
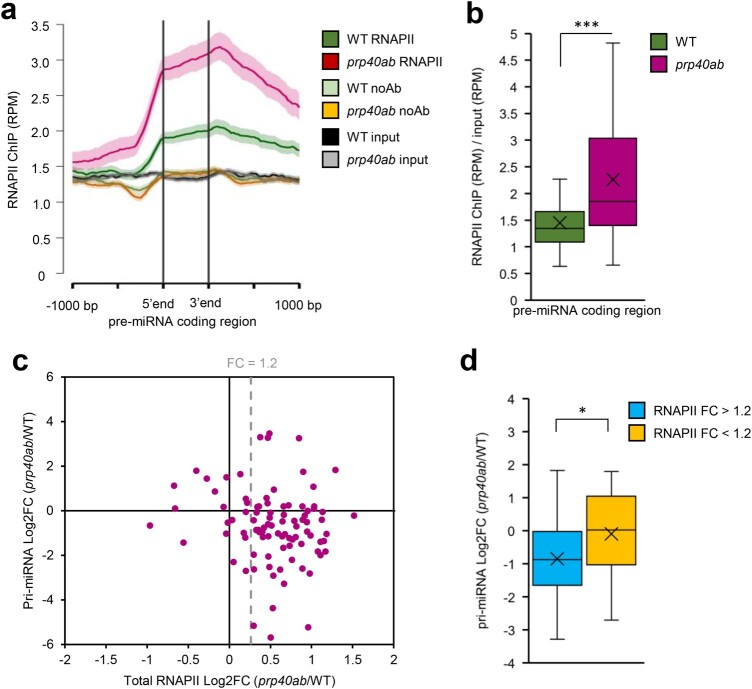
RNAPII distribution on miRNA genes is affected in the *prp40ab* mutant. A, Metagene analysis of RNAPII distribution on pre-miRNA coding regions based on ChIPseq data. B, Box plot showing the RNAPII occupancy on pre-miRNA coding regions based on ChIPseq data. C, Scatter plot showing the relationship between changes in RNAPII occupancy on pre-miRNA coding regions and changes in the levels of polyadenylated *MIR* transcripts in *prp40ab* plants compared with in WT plants. D, Box plot showing changes in the levels of polyadenylated *MIR* transcripts in *prp40ab* plants compared with in WT plants depending on a change in the RNAPII level in pre-miRNA coding regions. Mann–Whitney *U* test *P*-value: * < 0.05; *** < 0.001. Boxes are drawn between the first and third quartiles, with an additional line drawn along the second quartile to mark the median. “X” indicates the mean. Whiskers indicate the minimums and maximums outside the first and third quartiles. The shaded area around each curve on metaplots indicates standard errors.

The discordance between the levels of poly(A)-tailed pri-miRNAs and the total amount of *MIR* transcripts might be due to improper transcription termination and/or changes in miRNA precursor processing. We performed the whole-genome RNAPII profiling in the *prp40ab* mutant and compared the results obtained with those coming from WT plant analyses. The results revealed a higher level of RNAPII on *MIR*s in the *prp40ab* mutant, with a significant increase in pre-miRNA coding regions and further in 3′ ends (Figure 4, A and B; Supplemental Figures S14, a and c and S15a). Additionally, the accumulation of RNAPII along miRNA genes in *prp40ab* in most cases correlated with the reduced level of polyadenylated pri-miRNAs (Figure 4C), and the pri-miRNA coding regions with increased RNAPII occupancy had significantly lower levels of the corresponding poly(A)-tailed pri-miRNAs (Figure 4D).

Interestingly, the increased RNAPII occupancy was observed along protein coding genes as well, but polyadenylated transcript levels tested for selected genes (five intron-containing and five intronless genes) were not changed in the *prp40ab* mutant compared with in WT plants (Supplemental Figure S15, b and c).

To eliminate the possibility that PRP40 directly influences the initiation of transcription, we tested *MIR* promoter activity in *prp40ab* mutant plants by transfecting Arabidopsis protoplasts with constructs encoding luciferase under four different *MIR* promoters. We did not observe any significant difference in luciferase activity in the protoplast isolated from *prp40ab* mutant leaves when compared with the level of luciferase in transfected WT protoplasts (Supplemental Figure S16). This, together with the results showing no change of RNAPII occupancy on *MIR* transcription start sites (TSSs) in *prp40ab* (Supplemental Figure S15a), indicating that AtPRP40a and b proteins are not involved in *MIR* transcription initiation.

**Figure 5:**
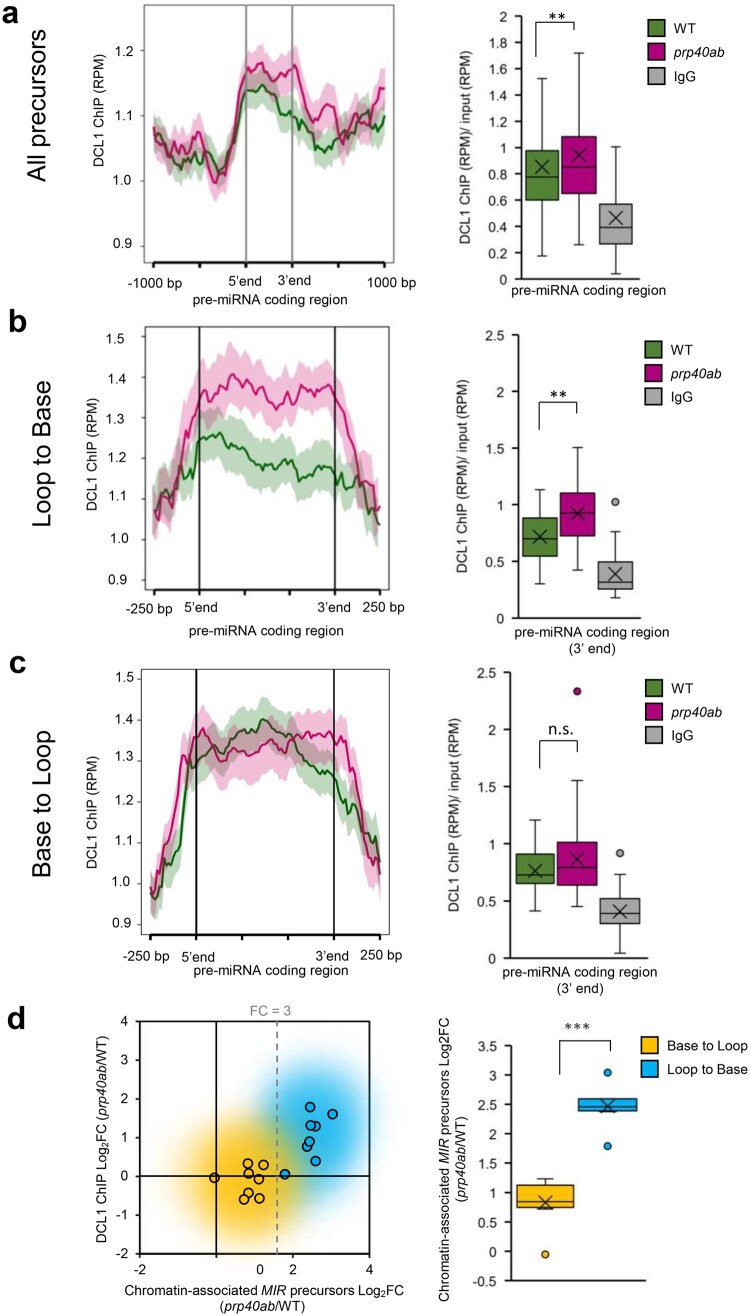
DCL1 distribution on *MIR* genes is affected in the *prp40ab* mutant. A, DCL1 distribution on pre-miRNA coding regions based on ChIPseq data. B, DCL1 distribution on LTB-type miRNA genes. C, DCL1 distribution on BTL-type miRNA genes. D, Relationship between a change in the level of chromatin-associated *MIR* transcripts and a change in the level of DCL1 on pre-miRNA coding regions depending on the miRNA gene type. Mann–Whitney *U* test *P-*value: ** < 0.01; *** < 0.001. Boxes are drawn between the first and third quartiles, with an additional line drawn along the second quartile to mark the median. “X” indicates the mean. Whiskers indicate the minimums and maximums outside the first and third quartiles. The shaded area around each curve on metaplots indicates standard errors.

Previously, DCL1 was detected on at least some *MIR*s (Fang et al., 2015); thus, we investigated how a lack of the AtPRP40a and b proteins affects DCL1 accumulation on chromatin. We observed alteration of the DCL1 level in pre-miRNA coding regions (Figure 5A and Supplemental Figure S14, b and c). On average, DCL1 occupancy was increased in the *prp40ab* mutant compared with in WT plants; however, there was a group of *MIR*s with unchanged or even decreased DCL1 levels. We recently showed that cotranscriptional processing of pri-miRNAs differs depending on the way precursors are cleaved at the first step (Gonzalo et al., 2022). For the LTB type of cleavage, both processing steps occur cotranscriptionally, whereas for the BTL type of processing, the first step is cotranscriptional but the second occurs post-transcriptionally in the nucleoplasm. We found that LTB-type *MIR*s, but not BTL-type *MIR*s, showed increased DCL1 occupancy in *prp40ab* mutant plants compared with in WT plants (Figure 5, B and C).

Both RNAPII and DCL1 ChIP-seq datasets strongly indicate that AtPRP40 proteins are involved in the cotranscriptional regulation of *MIR* expression. The increased RNAPII and/or DCL1 occupancy on pre-miRNA coding regions suggests that in *prp40ab* mutant plants, transcription and processing complexes are stuck on *MIR*s. To determine whether miRNA precursors also accumulated on *MIRs* in the *prp40ab* mutant plants compared with in the WT plants, we separated the nucleoplasmic and chromatin fractions of nuclei (Supplemental Figure S17a) and tested the miRNA precursor levels with RT-qPCR and primers amplifying the hairpin region of each precursor. In agreement with our previous data (Gonzalo et al., 2022), in the WT plants, we observed a high nucleoplasm/chromatin ratio of the hairpin fragments of *MIR* transcripts derived from BTL-type precursors (Supplemental Figure S17b). Furthermore, we calculated the *prp40ab*/WT fold change for chromatin and nucleoplasmic fractions and found high values (fold change over 3) for LTB-type precursors (Figure 5D and Supplemental Figure S17c), while BTL-type precursors showed only minor (fold change below 3) or no change in the chromatin fraction (Figure 5D and Supplemental Figure S17d). In the nucleoplasmic fraction, both types of precursors were mostly decreased in *prp40ab* plants compared with in WT plants. Interestingly, we did not find an increased chromatin association of full-length poly(A)-tailed precursors (Supplemental Figure S17e).

Finally, we measured the cotranscriptional processing rate for selected precursors with or without increased DCL1 occupancy in *prp40ab* plants. We compared the levels of the RT-qPCR products obtained with the use of RNA isolated from the chromatin fraction and primers hybridizing to the loop or hairpin region (these primers detected all precursors: unprocessed and processed) and the primers flanking the DCL1 cleavage site (these primers amplified only unprocessed precursors). We calculated the relative abundance of the loop/hairpin region over the amount of unprocessed pri-miRNAs and found lower values in *prp40ab* plants for precursors for which the DCL1 enrichment in *prp40ab* plants was observed (Supplemental Figure S18a). We did not observe any change for *MIR*s without increased DCL1 occupancy in *prp40ab* plants (Supplemental Figure S18b). These results demonstrate that overaccumulation of DCL1 on some pri-miRNAs does not lead to their more efficient processing.

Taken together, these results show that AtPRP40 regulates cotranscriptional miRNA biogenesis by affecting RNAPII and microprocessor activity on *MIR*s. This finding shows that AtPRP40 is involved in correct cotranscriptional microprocessor assembly.

## Discussion

Coupling of pre-mRNA processing with transcription is a well-established phenomenon (Greenleaf, 1993; McCracken et al., 1997; Fabrega et al., 2003; Braunschweig et al., 2013; Nojima et al., 2015, 2018). Cotranscriptional processing of miRNA precursors in humans has also been reported (Morlando et al., 2008; Yin et al., 2015). However, some results obtained demonstrate that mammalian pri-miRNA processing kinetics range from fast over intermediate to slow and that pri-miRNAs might be processed both co- and post-transcriptionally (Louloupi et al., 2017). Moreover, it has been claimed that chromatin retention does not determine the processing type, since the authors observed chromatin release of pri-miRNAs at comparable times after transcription for pri-miRNAs showing different processing kinetics. However, the factors regulating the kinetics of pri-miRNA processing and the co- and post-transcriptional mechanism of pri-miRNA processing have not been identified.

In human cells, the fused in sarcoma (FUS/translocated in liposarcoma) protein has been suggested to be a factor involved in the biogenesis of a large class of miRNAs, among which neuronal miRNAs are known to have a crucial role in neuronal function (Morlando et al., 2012). It has been shown that FUS is recruited to chromatin by binding to newly synthesized pri-miRNAs, where it facilitates Drosha (one of the two RNase III enzymes involved in miRNA biogenesis in animals) loading, supporting cotranscriptional processing of miRNA precursors (Morlando et al., 2012). It has also been suggested that the histone H1-like protein HP1BP3, but not canonical H1 variants, associates with the human microprocessor and promotes global miRNA biogenesis. HP1BP3 binds both DNA and pri-miRNA and enhances cotranscriptional miRNA processing via chromatin retention of nascent pri-miRNAs. This study clearly suggests the existence of a class of chromatin retention factors stimulating cotranscriptional miRNA processing in animal cells (Liu et al., 2016). Thus, the results indicate that both specific chromatin marks and additional protein factors connected with miRNA gene transcription may control the cotranscriptional assembly of the miRNA biogenesis machinery.

In contrast to that in animals, plant miRNA biogenesis occurs exclusively in the nucleus. Moreover, special nuclear structures called dicing bodies (D-bodies) are considered places of plant pri-miRNA processing (Fang and Spector, 2007). The fact that in each plant cell nucleus only a few D-bodies are observed may suggest a post-transcriptional mechanism of plant pri-miRNA processing and/or spatially clustering of *MIR*s near D-bodies. Recently, it was shown that phase separation of SE drives D-body formation and promotes miRNA processing (Xie et al., 2021). This SE phase separation might contribute to the association of D-bodies with chromatin. Recently, we have shown that pri-miRNA processing in plants is a cotranscriptional process; however, some steps occur post-transcriptionally in the nucleoplasm after release of the processing intermediates (miRNA-containing hairpins, pre-miRNAs) from the transcription sites. Moreover, we discovered that the structure of miRNA primary precursors dictates processing localization (Gonzalo et al., 2022). Plant pri-miRNAs were shown to be processed in two different manners: BTL (processing) and LTB (processing) (Zhu et al., 2013; Moro et al., 2018). We demonstrated that for pri-miRNAs processed in an LTB manner, both processing steps occur cotranscriptionally, and in the case of BTL-type pri-miRNA processing, the first step is cotranscriptional but the second occurs post-transcriptionally in the nucleoplasm. The data presented in this work confirm our previous conclusions on cotranscriptional miRNA biogenesis in plants (Gonzalo et al., 2022). We show here that BTL-type miRNA precursors accumulate predominantly in the nucleoplasm (Supplemental Figure S17b), in contrast to LTB-type transcripts that localize mostly on *MIR*s. In this article, we also identified a protein factor that is involved in the regulation of cotranscriptional miRNA biogenesis in plants. This protein is AtPRP40, the Arabidopsis U1 snRNP auxiliary protein.

The direct interaction between AtPRP40, which binds to the CTD of RNAPII, and the SE protein has been described by us previously (Knop et al., 2017). This observation prompted us to test whether SE forms a complex with RNAPII and whether the SE/RNAPII interaction requires the presence of AtPRP40. Indeed, we prove here that AtPRP40 mediates the association of SE with RNAPII (Figure 1, B and D and Supplemental Figure S7) on miRNA genes (Figure 2). Therefore, we further explored the role of AtPRP40 in miRNA biogenesis and cotranscriptional microprocessor assembly. We observed lower levels of polyadenylated miRNA precursors in the total RNA fraction (Figure 3A) but increased accumulation of nascent transcripts on *MIR*s in the *prp40ab* mutant plants (Supplemental Figure S17). We also found that RNAPII accumulates in the *prp40ab* mutant plants on pre-miRNA coding regions, which together indicate retention of RNAPII on *MIR*s when the AtPRP40a and b proteins are absent (Figure 4 and Supplemental Figure S14, a and c). The recruitment of DCL1 and SE, two key components of the plant microprocessor complex, to *MIR*s is also affected in *prp40ab* mutant plants, suggesting a role of AtPRP40 in cotranscriptional microprocessor assembly (Figures 2 and 5; Supplemental Figures S9 and S12, b and c). Thus, we added an additional element regulating DCL1 activity on miRNA genes to already published data on the role of Elongator, Mediator, and TREX-2 (Fang et al., 2015; Zhang et al., 2020; Cambiagno et al., 2021): AtPRP40 proteins and/or SE. All of these data point to a complex regulation of cotranscriptional microprocessor assembly at different steps of *MIR* transcription.

**Figure 6:**
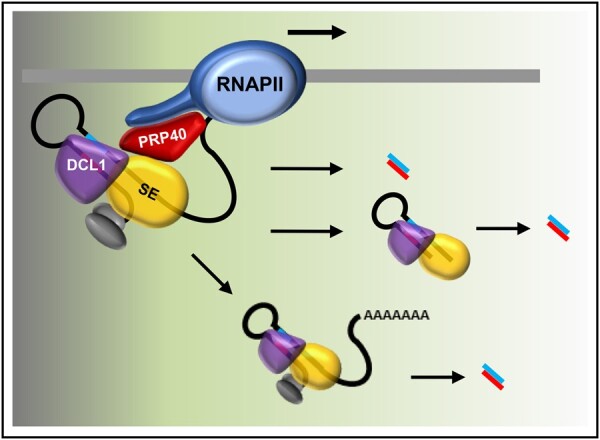
Cotranscriptional microprocessor assembly, regulated by AtPRP40, impacts RNAPII activity and is required for correct miRNA production. In WT plants, when AtPRP40 is present, SE and DCL1 are recruited to miRNA genes, primary precursors are efficiently processed to pre-miRNAs (hairpin structures) and further to miRNAs, and RNAPII fluently moves through the pre-miRNA coding region. In the case of LTB-type miRNA genes, both processing steps occur cotranscriptionally, and the miRNA/miRNA* duplex is released to the nucleoplasm. For BTL-type miRNA genes, only the first step is cotranscriptional, and the second cleavage step takes place in the nucleoplasm.

Moreover, our results indicate the existence of an interplay between the microprocessor and RNAPII. The influence of pre-mRNA processing on RNAPII activity was observed previously in the case of splicing. It has been shown that inactivation of the promoter proximal 5′ splice sites reduces the level of nascent transcription (Furger et al., 2002). Recently, it has also been reported that U2 snRNP has a positive effect on RNAPII transcription elongation (Caizzi et al., 2021). The existence of a transcriptional elongation checkpoint that is associated with cotranscriptional prespliceosome formation has been previously reported by the Beggs group (Chathoth et al., 2014). In Arabidopsis, we previously showed that NTR1, a spliceosome disassembly factor, is responsible for slowing RNAPII and the formation of splicing checkpoints at alternative splice sites (Dolata et al., 2015). Thus, similar to the interplay between the splicing and transcription machinery, we postulate here that correct microprocessor assembly, regulated by AtPRP40, has a positive effect on RNAPII transcription. In WT plants, AtPRP40 mediates the recruitment of SE to nascent *MIR* transcripts and facilitates the proper assembly of the whole microprocessor, processing of the primary miRNA precursors (Figure 6), and the smooth movement of RNAPII along pre-miRNA coding regions.

Interestingly, while RNAPII accumulates on most *MIR*s in *prp40ab* mutant plants, we observed increased occupancy of DCL1 only on approximately half of the *MIR*s in this mutant (Supplemental Figure S14c). On the rest of the *MIR*s, the accumulation of DCL1 in *prp40ab* mutant plants was not changed or decreased. DCL1 accumulation on *MIR*s in the *prp40ab* mutant is most likely due to the retention of nascent transcripts attached to the transcription sites. As already mentioned, both steps of processing of LTB pri-miRNAs are carried out cotranscriptionally, in contrast to BTL pri-miRNAs, where the second step of miRNA maturation takes place post-transcriptionally after releasing pre-miRNAs to the nucleoplasm. This difference in miRNA biogenesis can explain why the accumulation of DCL1 is observed only on LTB *MIR*s: stem–loop structures are cut out from BTL pri-miRNAs and released to the nucleoplasm, taking DCL1 away from the transcription sites. Additionally, DCL1 occupancy alternation might result in lower levels of polyadenylated miRNA precursors (but increased accumulation of non-polyadenylated precursors on *MIR*s in the *prp40ab* mutant plants). However, the higher association of DCL1 with *MIR*s and the retention of primary miRNA precursors on chromatin had no major effect on the levels of mature miRNAs (Figure 3B). In WT plants, cotranscriptional microprocessor assembly on newly synthesized pri-miRNAs stimulated RNAPII to pause the transcription of *MIR*s (Figure 4A). Impaired cotranscriptional microprocessor assembly leads to longer RNAPII pausing in the pre-miRNA coding region accompanied by the accumulation of miRNA precursors at their transcription sites. This decreases the levels of polyadenylated pri-miRNAs. Why the level of most miRNAs is not changed or is even slightly increased in *prp40ab* mutant plants is not clear. One possibility is that post-transcriptional processing of pri-miRNAs can compensate less efficient cotranscriptional processing of miRNA precursors at their transcription sites. We have demonstrated recently that pri-miRNAs can be processed both co- and post-transcriptionally and that the ratio between co- and post-transcriptional miRNA biogenesis can change at different environmental conditions (Gonzalo et al., 2022). Thus, it is possible that the levels of miRNAs that are processed mostly cotranscriptionally is complemented, when microprocessor assembly is impaired, by post-transcriptional processing of pri-miRNAs. However, this would require a special tunning mechanism to establish the right level of each miRNA in the cell, which have not been described yet. This phenomenon needs additional studies to confirm that the ratio between co- and post-transcriptional processing is different in *prp40ab* than in WT plants.

Our results demonstrate that AtPRP40 is the protein that contributes to the cotranscriptional recruitment of DCL1 and SE to pri-miRNAs, which regulate RNAPII activity over *MIR*s. However, we still do not exclude the possibility of more direct influences of AtPRP40 on transcription carried out by RNAPII. Additional studies are needed to distinguish between these possibilities.

## Materials and methods

### Plant material

Arabidopsis (*A. thaliana*; Col-0 WT, *se-1* [Prigge and Wagner, 2001], *se-2* [Grigg et al., 2005], *prp40a* [SALK_021070], *prp40b* [SALK_066044], *prp40c* [SALK_148319], *prp40ab* [SALK_021070 × SALK_066044], and *hyl1-2* [Vazquez et al., 2004]) seeds after 3 days of stratification were grown at 22°C (16-/8-h light/dark, 50%–60% humidity, 150–200 µmol m^−2^ s^−1^ photon flux density, Panasonic FL40SS ENW/37 fluorescent lamp 40W) in Jiffy-7 pots (Jiffy) and collected on day 21 of growth or used in crosses. The GFP-SE transgenic line was produced in the *se-4* (Lobbes et al., 2006) background by incorporation of an additional copy of SE fused with GFP under the control of the UBQ10 promoter. For ChIP analyses, 14-day-old seedlings grown on 0.5 Murashige and Skoog (MS) solid medium were used instead.

### RNA isolation and cDNA preparation

Total RNA for AtPRP40 mRNAs, pri-miRNA, and miRNA level analyses was prepared according to Dolata et al. (2021). Briefly, RNA was isolated using a Direct-zol RNA Mini Prep Kit (Zymo Research) and treated with Turbo DNase I (Thermo Fisher Scientific).

For transcript expression levels, 3 µg of total DNase-treated RNA was reverse transcribed to cDNA with the use of SuperScript III Reverse Transcriptase (Thermo Fisher Scientific) and oligo-dT(18) or random-hexamer primers (Thermo Fisher Scientific).

### Isolation of chromatin-bound and nucleoplasm RNA

Nascent transcripts and chromatin-associated RNAs were separated from the nucleoplasm using the protocol described by Conrad and Ørom (2017). cDNA was prepared with the use of random-hexamer primers or oligo(dT)_18_ (Thermo Fisher Scientific). RT-qPCR was performed using primers amplifying the hairpin region of *MIR* transcripts.

### RNA half-life measurements

Half-lives were determined as previously described (Seeley et al., 1992) with the following modifications. Arabidopsis seedlings were transferred into incubation buffer (Seeley et al., 1992), and 3′-deoxyadenosine (cordycepin; Sigma–Aldrich) was added to a final concentration of 0.6 mM (time 0). Tissue samples were harvested at regular intervals. Total RNA was isolated, treated with Turbo DNase (Thermo Fisher Scientific), and reverse-transcribed with oligo(dT)_18_ primer for qPCR. The experiment was performed in three biological replicates.

### RT-qPCR

RT-qPCR experiments were performed with Power SYBRR Green PCR Master Mix (Thermo Fisher Scientific) using a 7900HT Fast Real-Time PCR System (Thermo Fisher Scientific) as previously described (Knop et al., 2017). The primers from Supplemental Table S1 were used. Expression levels were calculated using the relative quantification (2^−ΔCt^), while the fold change was calculated using the 2^−ΔΔCt^ method (Dolata et al., 2021). The mRNA fragments of *GLYCERALDEHYDE-3-PHOSPHATE DEHYDROGENASE* (*GAPDH*, At1g13440) were amplified and detected simultaneously as a reference gene.

The Arabidopsis pri-miRNA expression platform mirEX 2.0 (Bielewicz et al., 2012; Zielezinski et al., 2015) was applied according to Dolata et al. (2021).

Each RT-qPCR was performed independently for three biological replicates. All results were analyzed using SDS 2.4 software (Thermo Fisher Scientific) and Microsoft Excel. Error bars were calculated using the SD Function in Microsoft Excel software. The statistical significance of the results presented was calculated using Student’s *t* tests at three significance levels: *P *<* *0.05, *P *<* *0.01, and *P *<* *0.001.

### Small RNA sequencing

Total RNA was isolated using the Direct-zol RNA kit (Zymo Research). RNA was quantified using a Qubit RNA Assay Kit (Life Technologies), and integrity was confirmed on an Agilent Bioanalyzer 2100 system. A total of 10 µg of each RNA sample was separated by electrophoresis on a 15% polyacrylamide 8 M urea gel in 1× TBE (Tris-borate-EDTA) buffer. Small RNA fractions were cut out and purified from the gel. Libraries were prepared using a TruSeq Small RNA Library Preparation Kit (Illumina). Single-end (1 × 50 bp) sequencing was performed at Fasteris, Geneva, Switzerland on a HiSeq 4000 platform. Adapter sequences were removed from raw reads with FASTX-Toolkit (fastx clipper). The clean reads were mapped to all mature Arabidopsis miRNAs found in miRBase (release 22) using countreads_mirna.pl script (Eminaga et al., 2013; Kozomara and Griffiths-Jones, 2014). The script was applied to each fastq file for every biological replicate. Statistical analysis was performed with the DESeq2 R package (Love et al., 2014).

### MiRNA processing precision calculation

The clean reads from small RNA sequencing were mapped to the Arabidopsis TAIR10 genome using Rsubread (Liao et al., 2019). The FeatureCount function from the RSubread package was used to obtain the number of reads of precisely processed miRNA (fracOverlapFeature = 1 and fracOverlap = 1 parameters) and imprecisely processed miRNA (fracOverlapFeature = 0 and fracOverlap = 0 parameters). In both cases, only uniquely mapped reads were counted (countMultiMappingReads = FALSE parameter). An annotation file from miRBase (release 22) was used for the mature miRNA coordinates. The processing precision value represents the ratio between precisely and imprecisely processed miRNAs.

### ChIP

ChIP was performed as described (Bowler et al., 2004) with IP buffer prepared as described (Kaufmann et al., 2010). Chromatin was sonicated at 4°C with a Diagenode Bioruptor Pico for ∼15 min (30 s on/30 s off) to obtain 250–500-bp DNA fragments. Antibodies against total RNAPII (Abcam ab817, 5 µg/IP), DCL1 (Agrisera AS19 4307, 10 µg/IP), and SE (Agrisera AS09 532A, 10 µg/IP) were used with Dynabeads Protein G (Thermo Fisher Scientific). For decrosslinking and DNA isolation, samples were treated with Proteinase K (Thermo Fisher Scientific) for 6 h at 55°C followed by purification with a Qiaquick PCR Kit (Qiagen). Libraries were prepared using a MicroPlex Library Preparation Kit (Diagenode) and sequenced on the NextSeq platform.

### Pull-down assay

The *E. coli* strain BL21-CodonPlus(De3)-RIL was transformed with pMal-derived plasmids encoding SE (full length or Δ681–720 aa) or AtPRP40b fused with maltose-binding protein and 6×His (MBP-6×His-SE/AtPRP40b). Overexpression was performed as follows: cells were grown for 16 h at 20°C after induction by 0.4 mM isopropyl β-d-1-thiogalactopyranoside and then harvested and sonicated (6 cycles of 45 s ON and 60 s OFF on ice) in lysis buffer (50 mM Tris–HCl pH 7.5, 300 mM NaCl, 10 mM imidazole, 5 mM β-mercaptoethanol, 0.5% Triton X-100, Roche Complete Mini EDTA-free protease inhibitor tablets [Sigma-Aldrich]). After sonication, lysates were centrifuged for 45 min at 8,000 × *g* at 4°C, and the supernatants containing the protein extract were collected. Proteins were purified with HisPur Ni-NTA Resin (Thermo Fisher Scientific), and MBP was cleaved off by TEV protease during overnight incubation in dialysis buffer (50 mM Tris–HCl pH = 7.5, 300 mM NaCl, 10 mM imidazole, and 5 mM β-mercaptoethanol) at 4°C. The TEV protease and MBP were removed in the additional purification step with the use of HisPur Ni-NTA Resin (Thermo Fisher Scientific). Next, SE variants and AtPRP40b were purified by size exclusion chromatography.

The biotinylated CTD peptides were synthesized by Thermo Fisher Scientific. For the pull-down experiment, Streptavidin MagneSphere Paramagnetic Particles (Promega) were washed three times with Buffer A (PBS pH 8.3, 5% glycerol, 1 mM DTT, 0.03% NP-40, Pierce Phosphatase Inhibitor Mini Tablets (Thermo Fisher Scientific)) and incubated with 2 µg of un, Ser5-, or Ser2-phosporylated CTD peptide for 2 h at 4°C in buffer A. Next, streptavidin particles with immobilized peptides were washed three times with buffer B (PBS pH 8.3, 5% glycerol, 1 mM DTT, 0.1% NP-40, Pierce Phosphatase Inhibitor Mini Tablets (Thermo Fisher Scientific), Roche Complete Mini EDTA-free protease inhibitor tablets (Sigma-Aldrich)), and incubated with 2 µg of SE variant, AtPRP40b, or both for 1.5 h at 4°C. Streptavidin particles were then washed five times with buffer B, and immobilized proteins were eluted with 3× Laemmli sample buffer (150 mM Tris–HCl pH 6.8, 150 mM DTT, 2% β-mercaptoethanol, 6% SDS, 0.03% bromophenol blue, 30% glycerol) at 25°C on a thermomixer (350 rpm). Protein samples were separated in a 10% sodium dodecyl sulfate-polyacrylamide gel, transferred to PVDF membranes, and detected by immunoblots. The following antibodies were used: anti-PRP40b (AS14 2785, Agrisera; 1:10,000), anti-His (sc-8036, Santa Cruz Biotechnology; 1:1,000), anti-rabbit (AS09 602, Agrisera; 1:20,000), and anti-mouse (sc-2005, Santa Cruz Biotechnology, 1:10,000). Input represents 1/10 of the protein sample.

### Luciferase activity

The sequence of *MIR* promoters (∼2.5 kb upstream of the gene start) was cloned into the 35Somega:Luc_nos (Martinho et al., 2015) plasmid in place of the 35S promoter (using BamHI and SalI restriction sites). The vector also contained the *Renilla* luciferase sequence taken from the 35SRLuc plasmid (Iwata and Koizumi, 2005).

The Arabidopsis protoplasts from WT and *prp40ab* plants were isolated according to Knop et al. (2017). The isolated protoplasts were transfected with 5 µg of DNA, and luciferase activity was measured 16 h after transfection with the use of Dual-Glo Luciferase Assay (Promega). The experiment was performed in three biological replicates.

### Immunoblot

Thirty micrograms of Arabidopsis whole leaf extract (extraction buffer: 100 mM Tris–HCl pH 7.5, 10% glycerol, 5 mM EDTA, 5 mM EGTA, 0.15 M NaCl, 0.75% Triton X-100, 0.05% SDS, and 1 mM DTT) was resolved in a 10% denaturing gel, transferred to PVDF membranes, and detected by immunoblotting. The following antibodies were used: anti-AtPRP40b (AS14 2785, Agrisera; 1:10,000), anti-DCL1 (AS19 4307; Agrisera; 1:100), anti-CBP80 (AS09 531; Agrisera: 1:2,000), anti-SE (AS09 532A; Agrisera; 1:2,000), anti-HYL1 (AS06 136; Agrisera: 1:1,000), anti-actin (691001, MP Biomedicals; 1:1,000), anti-rabbit (AS09 602, Agrisera; 1:20,000), and anti-mouse (sc-2005, Santa Cruz Biotechnology, 1:10,000).

### Immunolabeling and FISH

The experiment was performed on isolated nuclei of 35-day-old Arabidopsis leaves. The leaves were fixed in 4% paraformaldehyde in phosphate-buffered saline (PBS, pH 7.2) for 20 min and washed in 10 mM Tris–HCl (pH 7.5). Nuclei isolation was performed according to the method described in Pontvianne et al. (2016). The nuclei were permeabilized with PBS + 0.1% Triton X-100 for 10 min. The following primary antibodies were applied according to Bhat et al. (2020): anti-SE (AS09 532A; Agrisera, 1:100), anti-PRP40b (AS14 2785; Agrisera; 1:100), anti-RNAPII-CTD-Ser5 (3E8; Chromotek; 1:100), and anti-RNAPII-CTD-Ser2 (3E10; Chromotek; 1:100). For the localization of GFP-SE, we used mouse antibodies targeting GFP (ab1218; Abcam; 1:100). Primary antibody incubation (in 0.01% acetylated BSA in PBS) was performed in a humidified chamber overnight at 11°C. After PBS washes, the slides were incubated with the following secondary antibodies: anti-rabbit Alexa Fluor plus 555 (A32732; Thermo Fisher Scientific; 1:200) and anti-rat Alexa Fluor 488 (A-11006; Thermo Fisher Scientific; 1:200) or anti-mouse Alexa Fluor Plus 488 (A32723; Thermo Fisher Scientific; 1:200). The secondary antibodies were diluted in PBS + 0.01% acetylated BSA and incubated at 37°C in a humidified chamber for 1 h.

In double-labeling FISH-immunofluorescence reactions (U1 snRNA + AtPRP40b protein), the in-situ hybridization method always preceded the immunocytochemical method. Prior to FISH, the nuclei were permeabilized with PBS + 0.1% Triton X-100. The probe targeting U1 snRNA was labeled at the 5′-end with digoxigenin and was resuspended in hybridization buffer (30%, v/v, formamide, 4× SSC, 5× Denhardt’s buffer [0.1% Ficoll 400, 0.1% polyvinylpyrrolidone, and 0.1% bovine serum albumin], 1 mM EDTA, and 50 mM phosphate buffer) at a concentration of 50 pmol/mL. Hybridization was performed overnight at 28°C. Digoxygenin (DIG) probes were detected after hybridization using mouse anti-DIG (11333062910; Merck; 1:100) and anti-mouse Alexa Fluor 488 (A-11001; Thermo Fisher Scientific, 1:200) antibodies in 0.01% acetylated BSA in PBS.

The slides were stained for DNA detection with Hoechst 33342 (Life Technology) and mounted in ProLong Gold antifade reagent (Life Technologies, P36934).

Correlation analysis was performed with Pearson’s correlation coefficient, Spearman’s rank correlation, and the ICQ value. We also used Colocalization Colormap according to Jaskolski et al. (2005). The statistical analysis was performed using Fiji plugins: coloc2 and Colocalization Colormap (Jaskolski et al., 2005; Dobbyn et al., 2018). The obtained results were analyzed by Student’s *t* tests at *P *<* *0.001 with *n* = 50 (proteins co-localization) or 30 (co-localization of U1 snRNA and AtPRP40b). The most representative images are shown.

### Proximity ligation assay

PLA detection was performed using a Duolink In Situ Orange Kit (Merck) according to the manufacturer’s protocol. Prior to the method, the nuclei were treated with PBS buffer containing 0.1% Triton X-100 and then incubated with Duolink blocking solution at 37°C in a humidified chamber for 60 min. After washing, a two-stage protocol was applied with the following antibodies: primary rabbit antibodies recognizing SE (AS09 532A, Agrisera, 1:100) and AtPRP40b (AS14 2785, Agrisera, 1:100), rat antibodies for the detection of phosphorylated RNAPII (serine 5 and serine 2) (Chromotek, 1:100), and secondary goat anti-rat Alexa Fluor 488 antibodies (A-11006, Thermo Fisher Scientific, 1:200). The antibodies were diluted in PBS buffer containing 0.05% acetylated BSA, and the incubation was performed overnight at 10°C (primary antibodies) or at 37°C for 2 h. After incubation, the nuclei were washed with wash buffer A and subjected to incubation with the Duolink anti-rabbit PLA-plus probe and the Duolink anti-goat PLA-minus probe in Duolink antibody diluent (diluted to 1:40) at 37°C for 1 h. Next, after washing, the slides were incubated with the ligation mix containing ligase at 37°C for 30 min. Furthermore, amplification buffer containing polymerase was applied. The amplification reaction was performed for 100 min at 37°C. This type of two-stage protocol allows the determination of localization of both the total pool of RNAPII (green fluorescence) as well as those that are associated with SE or AtPRP40b (red spots of fluorescence). The slides were stained for DNA detection with Hoechst 33342 (Life Technology) and mounted in ProLong Gold antifade reagent (Life Technologies, P36934). Twenty different cells were captured, and the most representative image is shown.

### Microscopy

The obtained results were registered with a Leica SP8 confocal microscope using a diode 405 laser, an argon/ion laser with a wavelength of 488 nm and a diode laser DPSS 561 that emitted light with a wavelength of 561 nm. For an optimized pinhole, a long exposure time (200 kHz) and 63× (numerical aperture, 1.4) Plan Apochromat DIC H oil immersion lens were used. Images were collected sequentially in blue (Hoechst 33342), green (Alexa 488 fluorescence), and red (Alexa 555, PLA Orange) channels. For bleed-through analysis and control experiments, Leica SP8 software was used.

## Accession numbers

The data reported in this article have been deposited in the NCBI GEO database, https://www.ncbi.nlm.nih.gov/geo (accession no. GSE187461).

## Supplemental data

The following materials are available in the online version of this article.

**Supplemental Figure S1.** *AtPRP40* genes are differentially expressed during Arabidopsis development.

**Supplemental Figure S2.** AtPRP40b is weakly colocalized with U1 snRNA.

**Supplemental Figure S3.** AtPRP40 is important for Arabidopsis development.

**Supplemental Figure S4.** Redundant role of the AtPRP40a and b proteins.

**Supplemental Figure S5.** Crosstalk between SE and AtPRP40 is crucial for plant development.

**Supplemental Figure S6.** AtPRP40b colocalizes with SE.

**Supplemental Figure S7.** AtPRP40 regulates the colocalization of SE and RNAPII.

**Supplemental Figure S8.** Colocalization of AtPRP40b and RNAPII in the cell nucleus is not regulated by SE.

**Supplemental Figure S9.** AtPRP40 is required for the proper accumulation of SE on miRNA genes.

**Supplemental Figure S10.** Effect of AtPRP40 on miRNA biogenesis.

**Supplemental Figure S11.** PRP40 does not affect the stability of poly(A)-tailed pri-miRNAs.

**Supplemental Figure S12.** Single *prp40a/b/c* mutants do not show changes in the pri-miRNA levels.

**Supplemental Figure S13.** Levels of miRNA biogenesis-related proteins in *prp40ab.*

**Supplemental Figure S14.** RNAPII and DCL1 distributions on *MIR* genes are affected in the *prp40ab* mutant.

**Supplemental Figure S15.** RNAPII distribution is affected in the *prp40ab* mutant.

**Supplemental Figure S16.** *MIR* promoter activity does not depend on AtPRP40.

**Supplemental Figure S17.** Distribution of *MIR* gene transcripts between chromatin and the nucleoplasm is affected in the *prp40ab* mutant.

**Supplemental Figure S18.** Cotranscriptional processing rate in *prp40ab* mutant plants depends on DCL1 distribution on *MIR* genes.

**Supplemental Table S1.** Primers used in this study.

**Supplemental Table S2.** *T* test results.

## Supplementary Material

koac278_Supplementary_Data

## Notes

A.S., J.D., A.J., and Z.S.-K. designed the research. A.S., J.D., T.G., D.B., M.B., and D.J.S. performed the research. A.S., J.D., T.G., D.B., M.B., D.J.S., Z.S.-K., and A.J. analyzed the data and wrote the manuscript.

The author responsible for distribution of materials integral to the findings presented in this article in accordance with the policy described in the Instructions for Authors (https://academic.oup.com/plcell) is: Artur Jarmolowski (artjarmo@amu.edu.pl).
