# Supplementary material for: Chromatin-associated microprocessor assembly is regulated by the U1 snRNP auxiliary protein PRP40
Source: Plant Cell. 2022 Sep 10;34(12):4920–35. doi: 10.1093/plcell/koac278 (PMC9709975; doi:10.1093/plcell/koac278)
Supplement: koac278_Supplementary_Data [file koac278_supplementary_data.pdf]

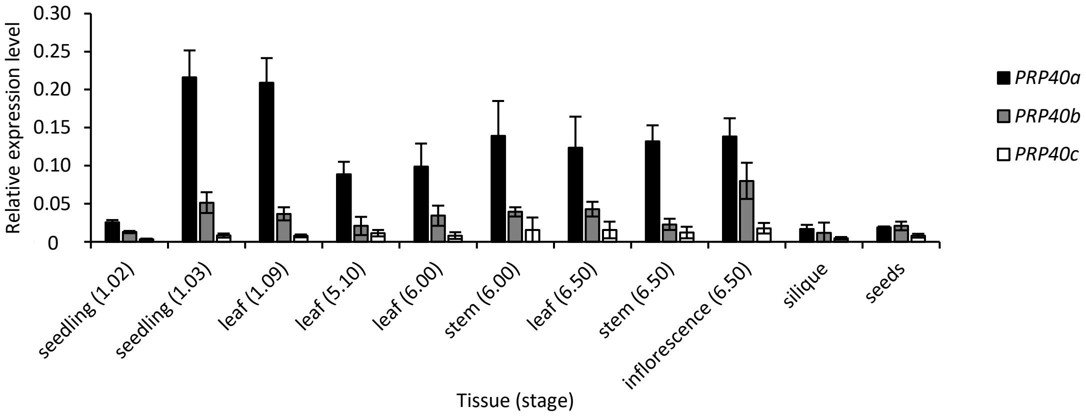

Supplemental Figure S1 *AtPRP40* genes are differentially expressed during Arabidopsis development. RT-qPCR showing differences in the expression of the *AtPRP40A*, *B* and *C* genes in different Arabidopsis tissues and growth stages. Numbers in brackets on the x-axis indicate growth stages according to Boyes et al. 2001. Data represent means ± SD (n = 3). Supports Figure 1.

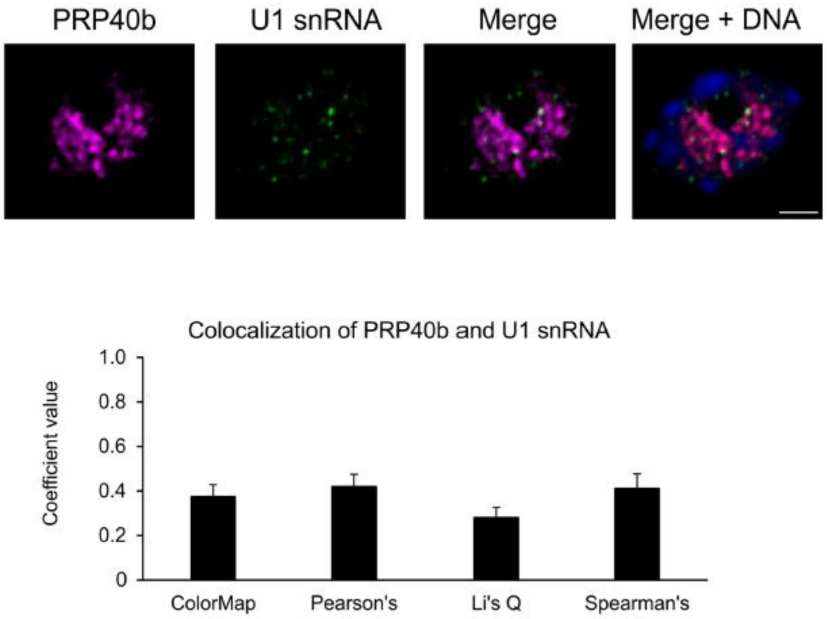

Supplemental Figure S2 AtPRP40b is weakly colocalized with U1 snRNA. Colocalization of AtPRP40b (first image, magenta signals) and U1 snRNA (second image, green signals) in the cell nucleus. DNA was stained with Hoechst (blue). Scale bar = 2.5 µm (*upper panel*). Colocalization coefficient values of the colocalization of AtPRP40b and U1 snRNA are shown below the images (*lower panel*). Data represent means ± SD (n = 4). Supports Figure 1.

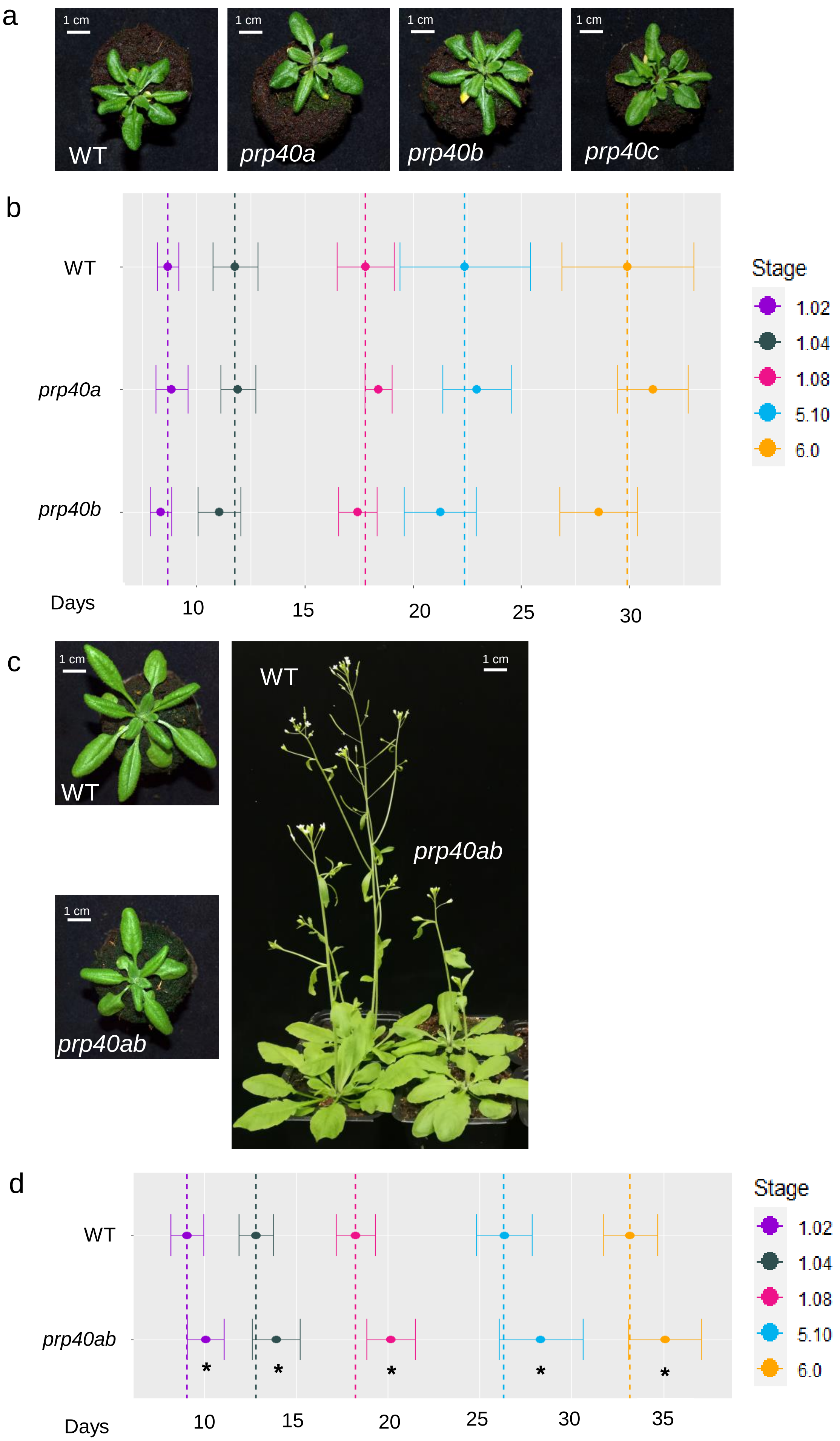

Supplemental Figure S3 AtPRP40 is important for Arabidopsis development. A, Phenotypes of the *prp40a, prp40b* and *prp40c* single mutants and the wild type (WT). b, Growth analysis of the *prp40a, prp40b* and *prp40c* single mutants and the WT according to Boyes et al. 2001. Data represent means ± SD (n = 3). c, Phenotype of the *prp40ab* double mutant and the WT. d, Growth analysis of the *prp40ab* mutant and the WT according to Boyes et al. 2001. Data represent means ± SD (n = 3). * $P < 0.05$. Supports Figure 1.

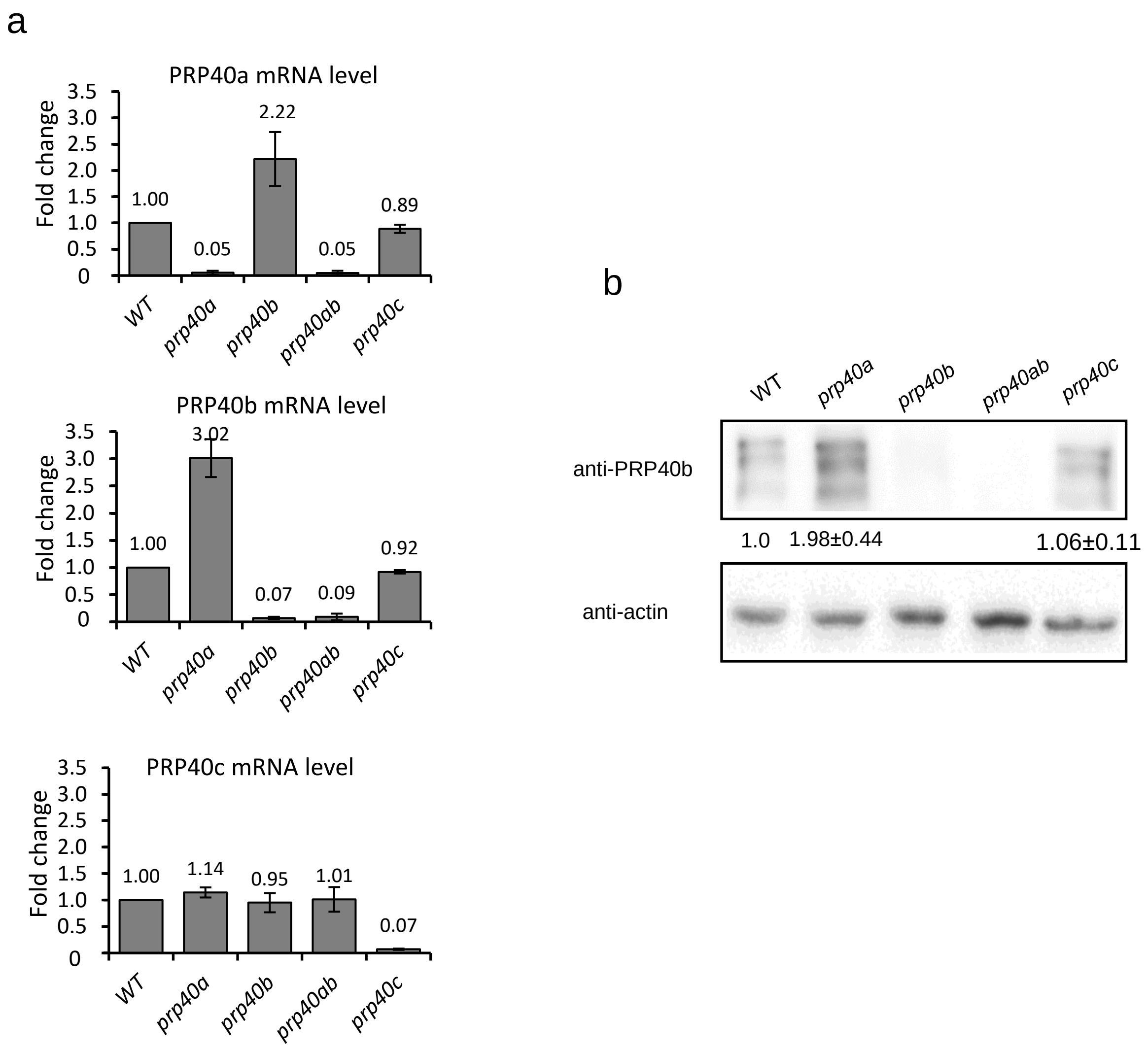

Supplemental Figure S4 Redundant role of the AtPRP40a and b proteins. A, RT-qPCR showing the expression of *AtPRP40* genes in the single and double *prp40* mutants and the wild type (WT). Data represent means ± SD (n = 3). b, Immunoblot showing AtPRP40b protein levels in the single and double *atprp40* mutants and the WT. Numbers below the immunoblot represent means ± SD (n = 3). Supports Figure 1.

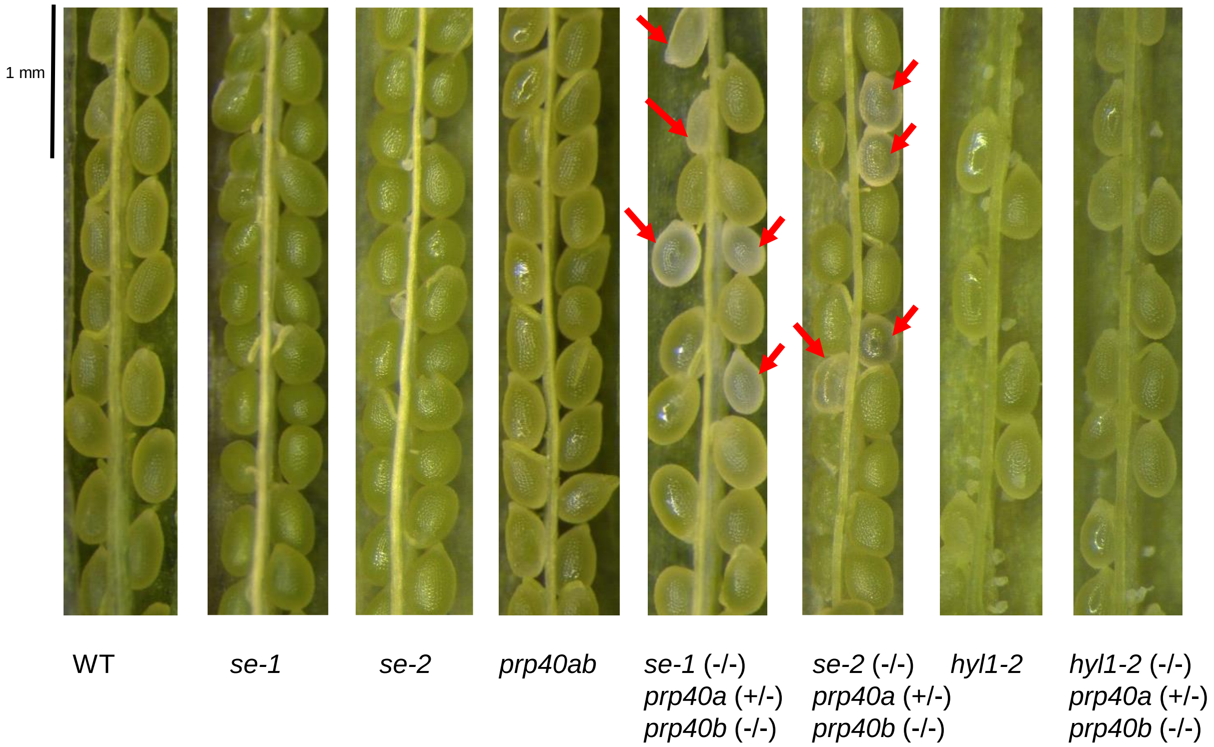

Embryos	WT	*se-1*	*se-2*	*prp40ab*	*se-1xprp40ab*	*se-2xprp40ab*	*hyl1-2*	*hyl1-2xprp40ab*
Counted	188	215	99	205	212	283	35	209
% abnormal	2.75	6.56	2.08	7.75	25.69	26.37	0.98	3.26
SD	4.24	5.57	2.95	5.37	9.31	7.23	0.05	6.30

Supplemental Figure S5 Crosstalk between SE and AtPRP40 is crucial for plant development. Abnormal, developing embryos are indicated by red arrows. The number and percentage of abnormal embryos for each genotype are presented in the table. Aborted ovules in *hyl1-2* and *prp40ab* × *hyl1-2* were excluded from the analysis. Data represent means ± SD. N = 3 biological replicates with ≥ 3 siliques in each biological replicate). WT, wild type. Supports Figure 1.

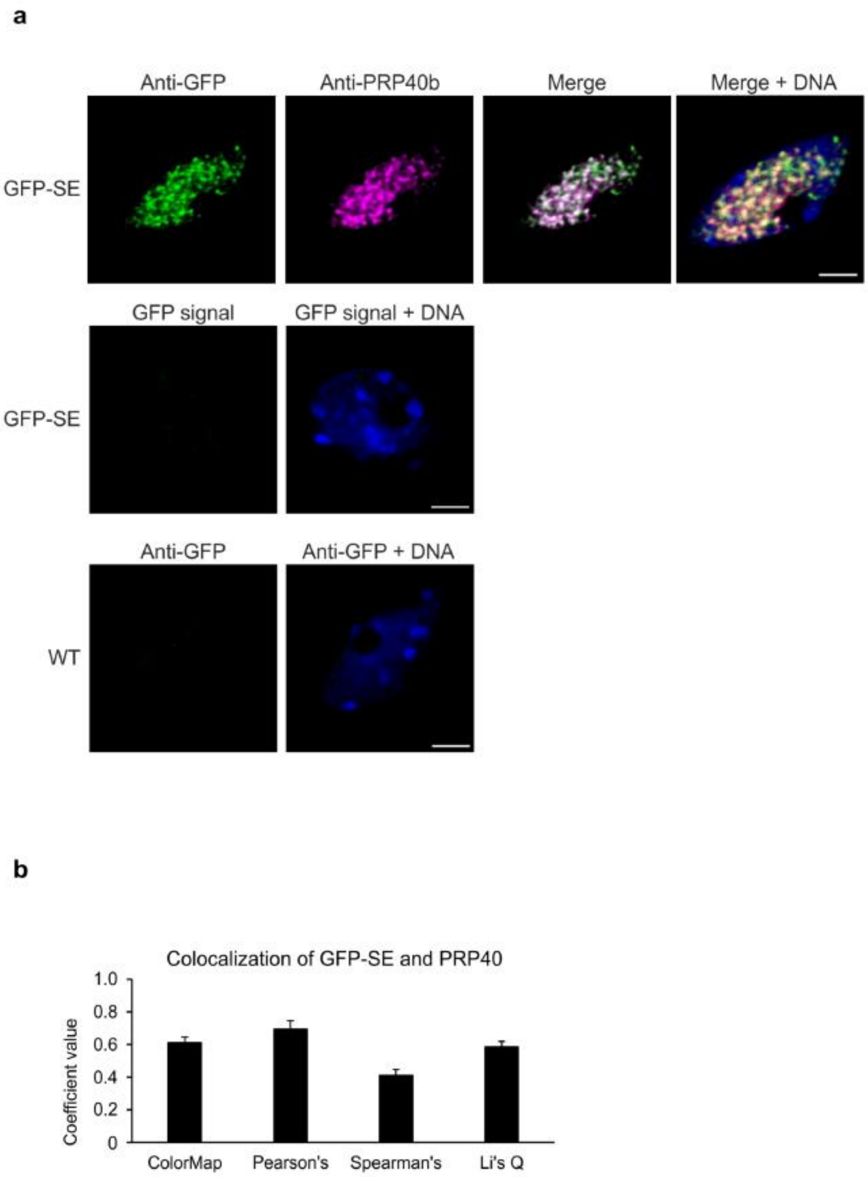

Supplemental Figure S6 AtPRP40b colocalizes with SE. a, Colocalization of SE (first image, magenta signals) and AtPRP40b (second image, green signals) in the cell nucleus of the Arabidopsis transgenic line expressing GFP-SE. “GFP signal” indicates the signal recorded with the 488 nm laser on a fixed sample prepared without the use of any antibodies. DNA was stained with Hoechst (blue). Scale bar = 2.5 µm. b, Coefficient values for colocalization of GFP-SE and PRP40b in the cell nucleus. Data represent means ± SD. N = 3. Supports Figure 1.

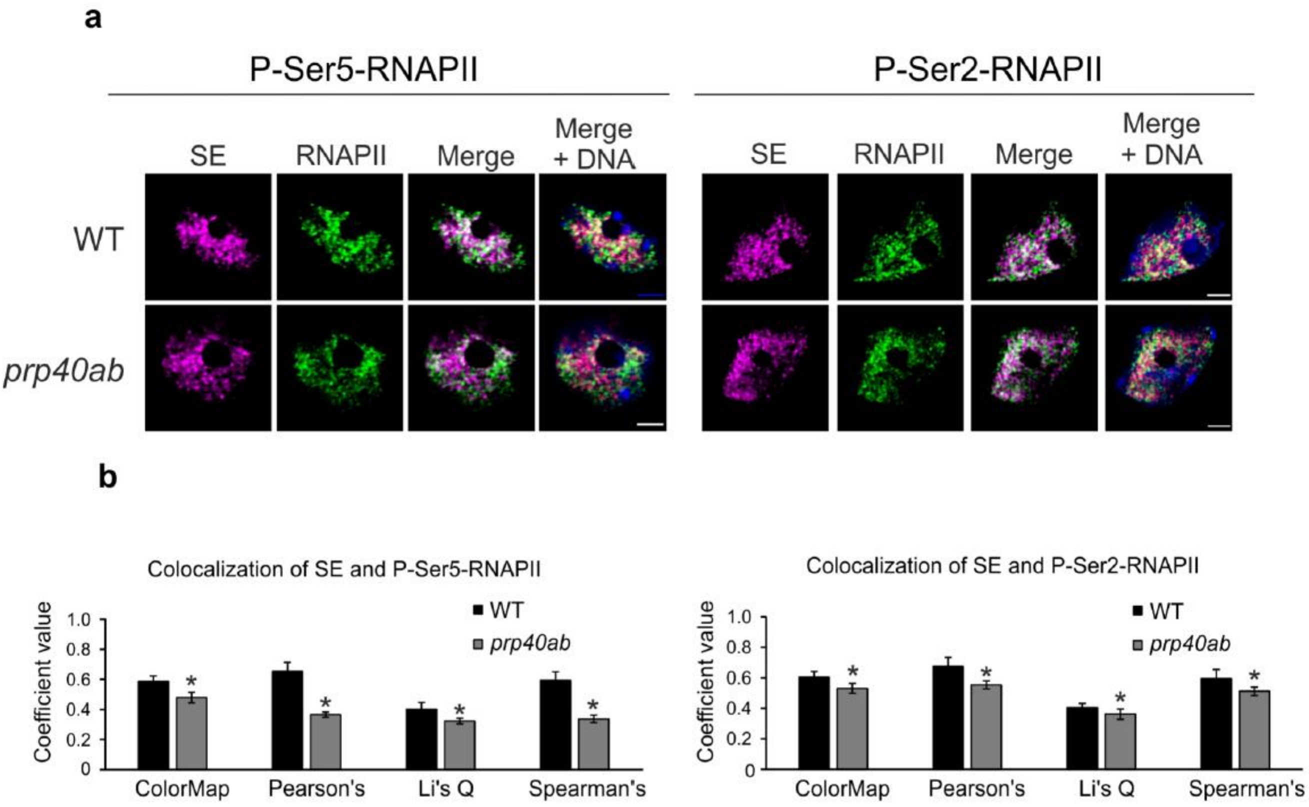

Supplemental Figure S7 AtPRP40 regulates the colocalization of SE and RNAPII. a, Colocalization of SE (first column, magenta signals) and RNAPII phosphorylated at CTD Ser5 (P-Ser5-RNAPII) or Ser2 (P-Ser2-RNAPII) (second column, green signals) in wild-type (WT) and *prp40ab* cell nuclei. DNA was stained with Hoechst (blue). Scale bar = 2.5 μm. b, Coefficient values for the colocalization of SE and RNAPII CTD phosphorylated at CTD Ser5 (P-Ser5-RNAPII) or Ser2 (P-Ser2-RNAPII) in WT and *prp40ab* cell nuclei. Data represent means ± SD. N = 3. * $P < 0.001$ Supports Figure 1.

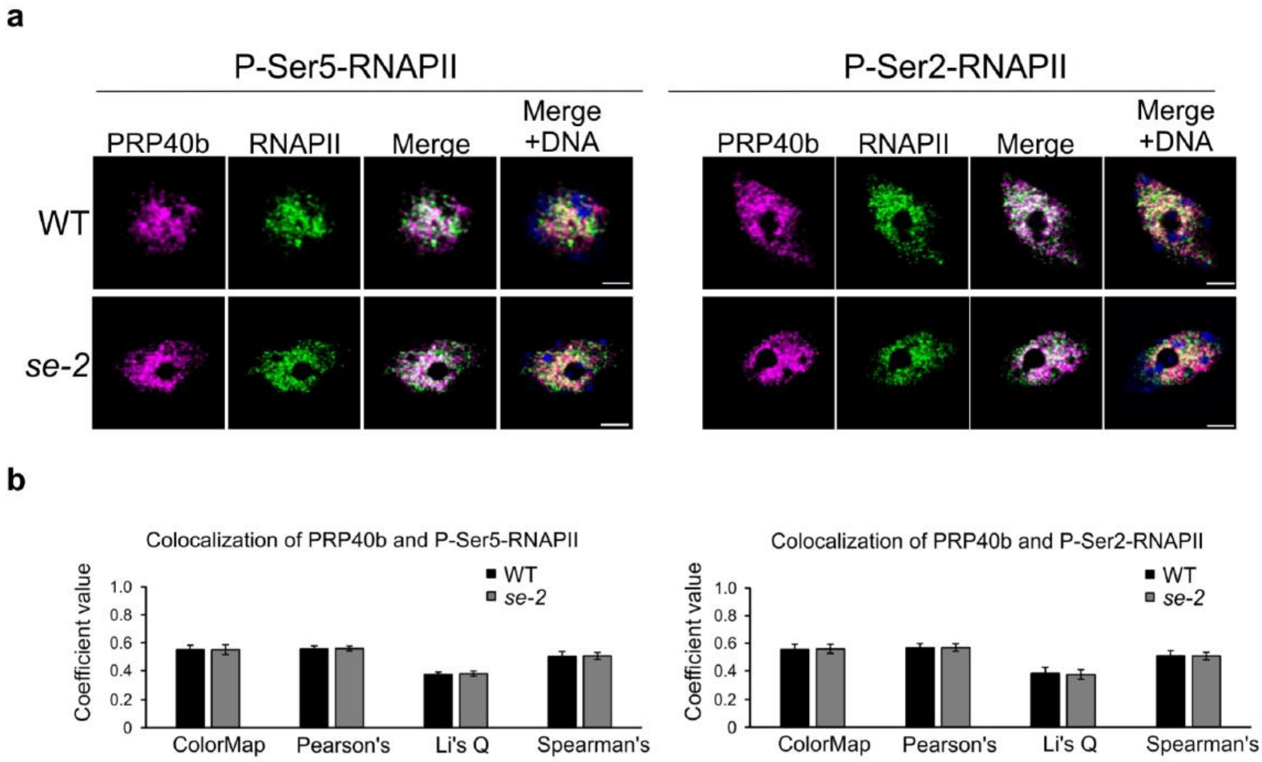

Supplemental Figure S8 Colocalization of AtPRP40b and RNAPII in the cell nucleus is not regulated by SE. a, Colocalization of AtPRP40b (first columns, magenta signals) and RNAPII phosphorylated at CTD Ser5 (P-Ser5-RNAPII) or Ser2 (P-Ser2-RNAPII) (second columns, green signals) in wild-type (WT) and *se-2* cell nuclei. DNA was stained with Hoechst (blue). Scale bar = 2.5 µm. b, Coefficient values for the colocalization of AtPRP40b and RNAPII CTD phosphorylated at CTD Ser5 (P-Ser5-RNAPII) or Ser2 (P-Ser2RNAPII) in WT and *se-2* cell nuclei. Data represent means ± SD. N = 3. * $P < 0.001$. Supports Figure 1.

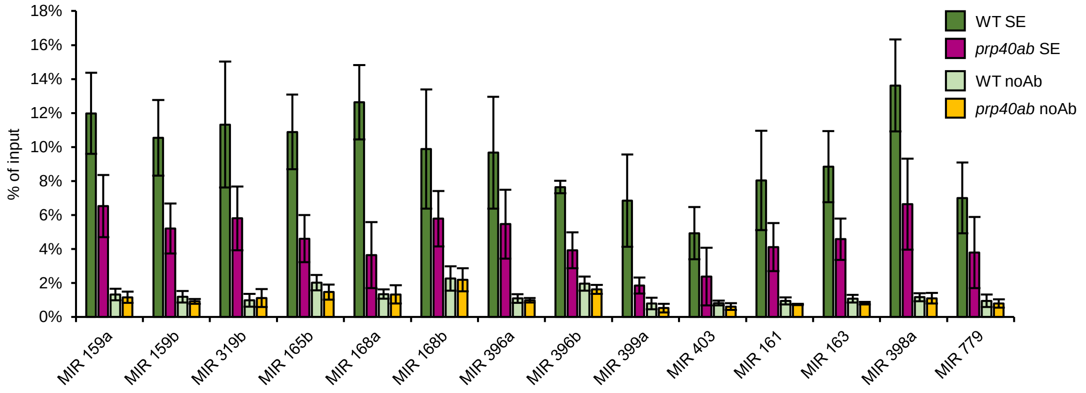

Supplemental Figure S9 AtPRP40 is required for the proper accumulation of SE on miRNA genes. Quantitative ChIP-PCR showing the level of SE on miRNA genes in the *prp40ab* mutant and the wild type (WT). Data represent means ± SD (n = 3). Primers amplifying pri-miRNA/pre-miRNA coding regions were used. Supports Figure 2.

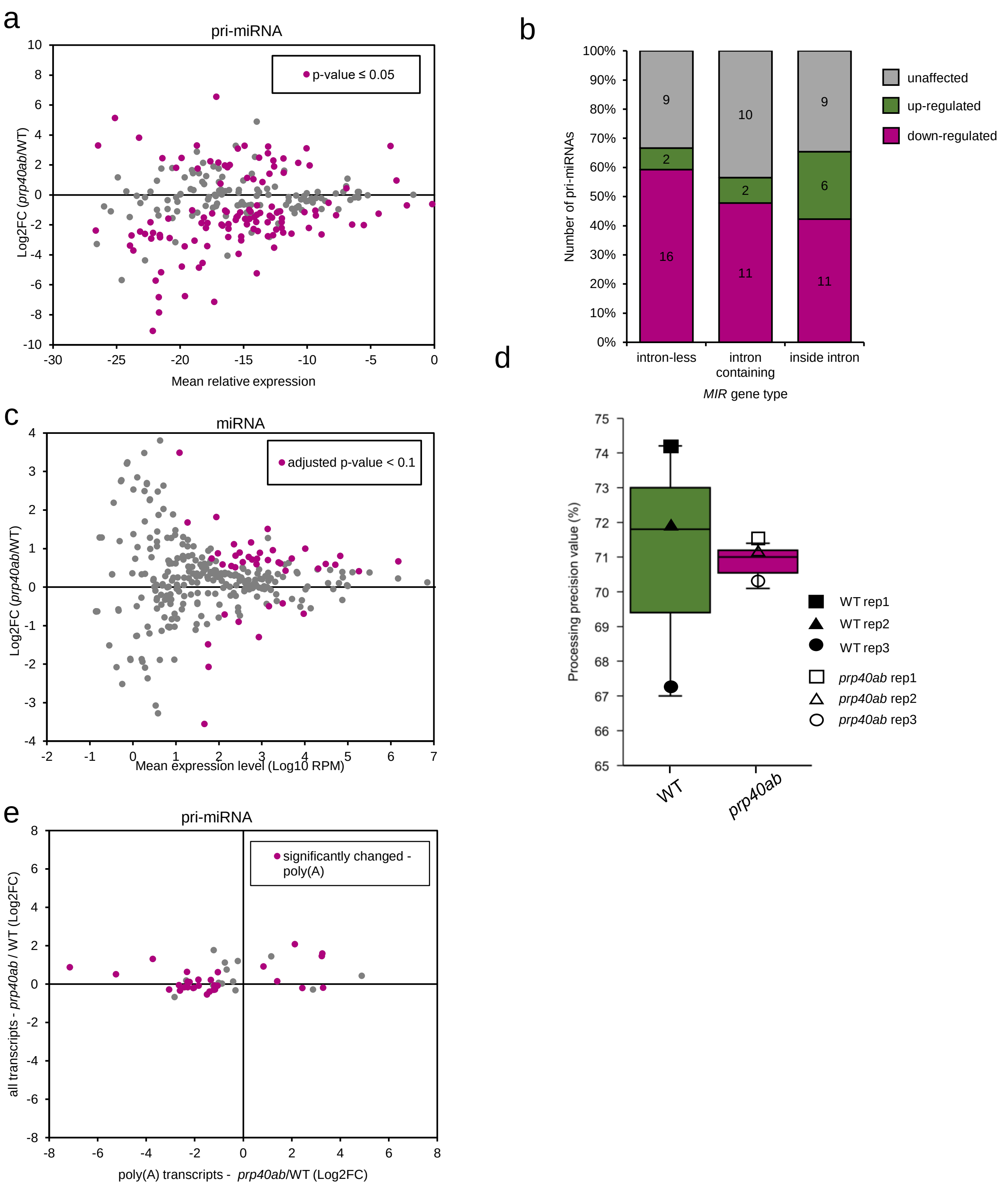

Supplemental Figure S10 Effect of AtPRP40 on miRNA biogenesis. a, MA plot showing a relation between the fold change (*prp40ab*/wild type (WT)) of polyadenylated pri-miRNA and its mean relative expression. b, Number of changed polyadenylated pri-miRNAs in *prp40ab* depending on the miRNA gene type. c, MA plot showing a relation between the fold change (*prp40ab*/WT) of miRNA and its mean relative expression. d, Box plot showing the precision of miRNA processing in *prp40ab* plants compared with in WT plants. Boxes are drawn between the first and third quartiles, with an additional line drawn along the second quartile to mark the median. Whiskers indicate the minimums and maximums outside the first and third quartiles. e, Fold change (*prp40ab*/WT) comparison for polyadenylated and total *MIR* transcripts. Supports Figure 3.

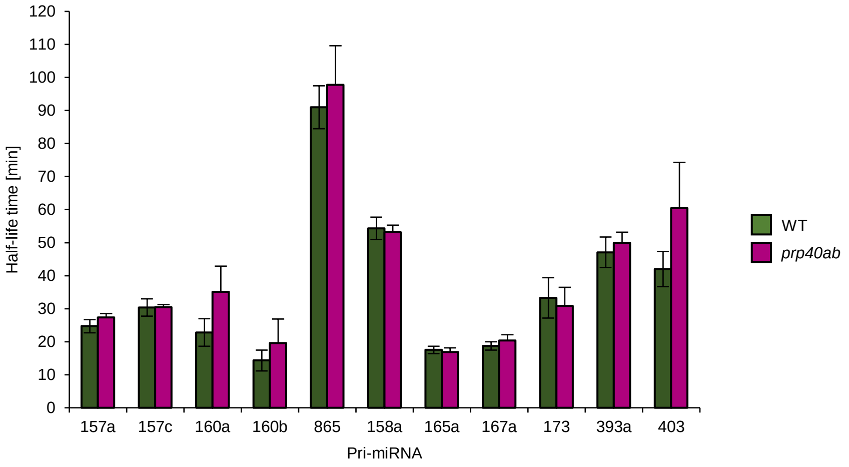

Supplemental Figure S11 PRP40 does not affect the stability of poly(A)-tailed pri-miRNAs. Half-life time measurements using RT-qPCR were conducted after blocking transcription with cordycepin. Data represent means ± SD (n = 3). WT, wild type. Supports Figure 3.

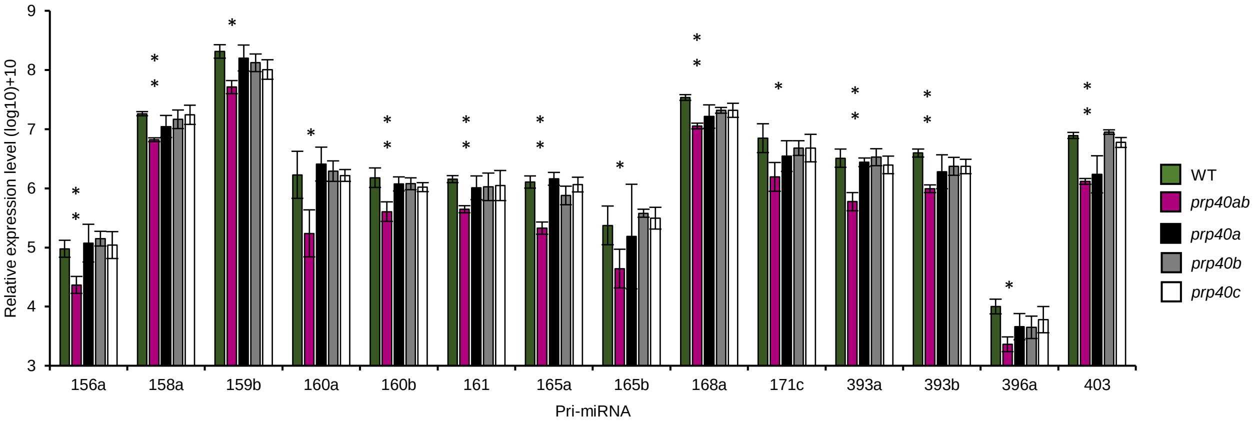

Supplemental Figure S12 Single *prp40a/b/c* mutants do not show changes in the pri-miRNA levels. RT-qPCR showing relative expression levels of selected pri-miRNAs that were downregulated in the *prp40ab* double mutant. WT, wild type. Data represent means ± SD. Means were compared using *t*-tests; *P*-value: * < 0.05; ** < 0.01 (n = 3). Supports Figure 1.

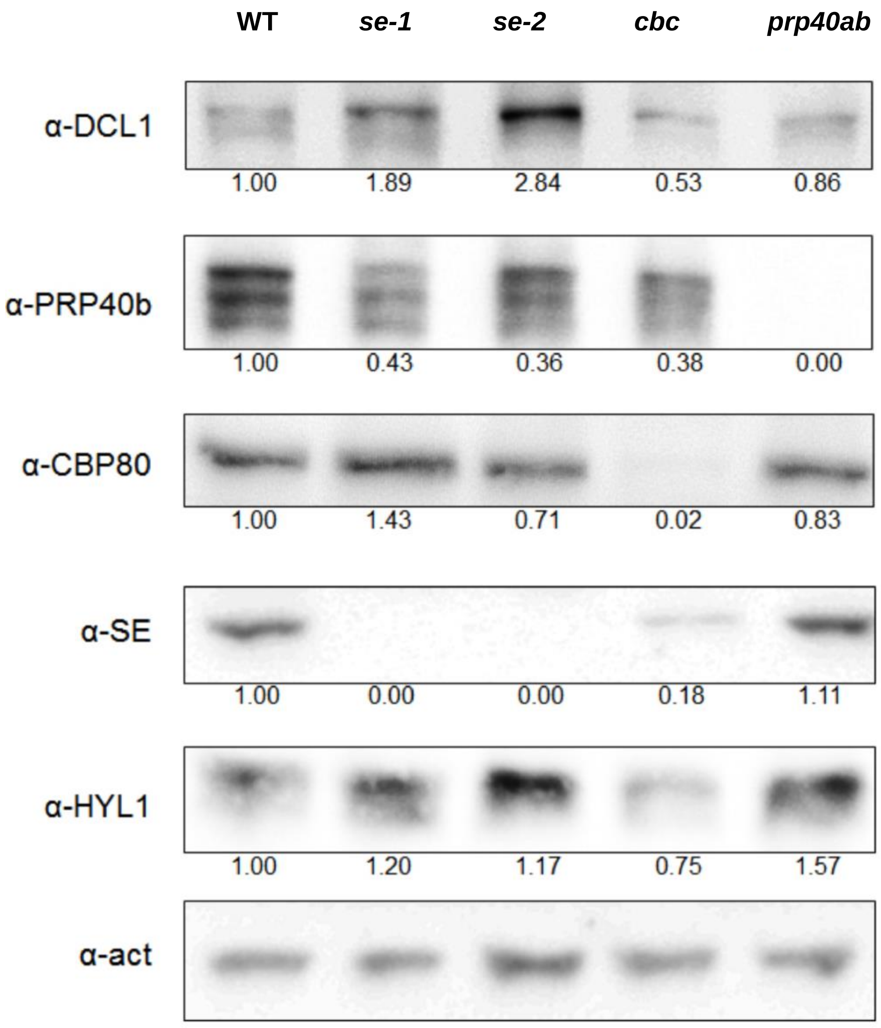

Supplemental Figure S13 Levels of miRNA biogenesis-related proteins in *prp40ab.* DCL1, SE, HYL1, CBP80 and PRP40b levels in the *se-1*, *se-2*, *cbp20* × *cbp80* and *prp40ab* mutants and in the wild type (WT) determined by immunoblots. The level of ACTIN was used as a loading and normalization control. The numbers below the blots represent quantification of the band intensities. Supports Figure 1

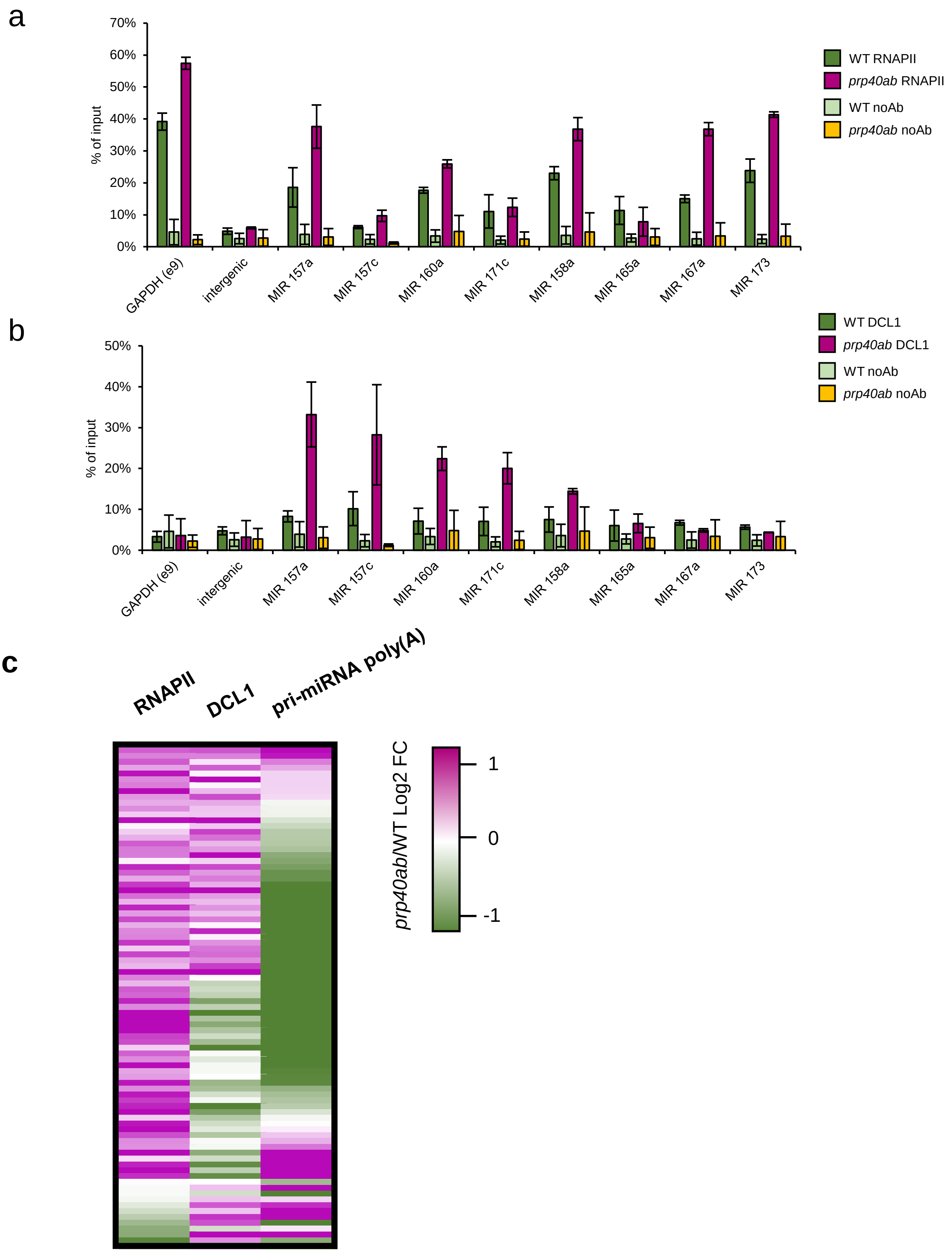

Supplemental Figure S14 RNAPII and DCL1 distributions on *MIR* genes are affected in the *prp40ab* mutant. a, ChIP-qPCR showing changes in the RNAPII distribution on *MIR* genes in the *prp40ab* mutant and the wild type (WT). Data represent means ± SD (n = 2). b, ChIP-qPCR showing changes in the DCL1 distribution on *MIR* genes in the *prp40ab* mutant and the WT. Data represent means ± SD (n = 2). c, Heatmap based on RNAPII ChIPseq, DCL1 ChIPseq and RT-qPCR data for miRNA precursors. Only precursors with data available from three experiments are shown. Supports Figure 4 and 5.

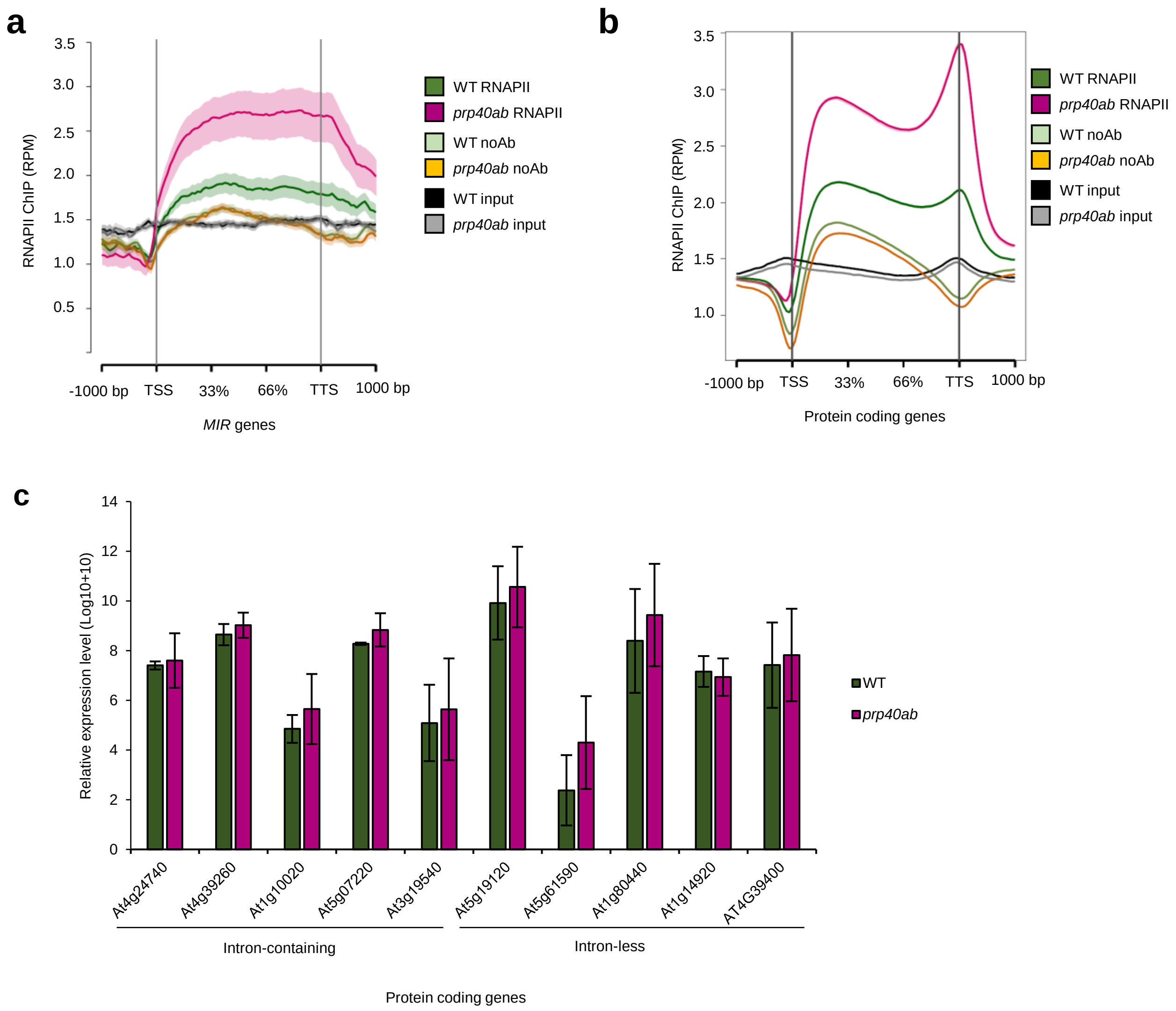

Supplemental Figure S15 RNAPII distribution is affected in the *prp40ab* mutant. a, Metagene analysis of RNAPII distribution on miRNA genes with known structure based on ChIPseq data b, Metagene analysis of RNAPII distribution on protein coding genes based on ChIPseq data c, RT-qPCR showing relative expression levels of randomly selected protein coding genes with increased RNAPII occupancy in the *prp40ab* mutant and the wild type (WT). Data represent means ± SD (n = 3). Supports Figure 4.

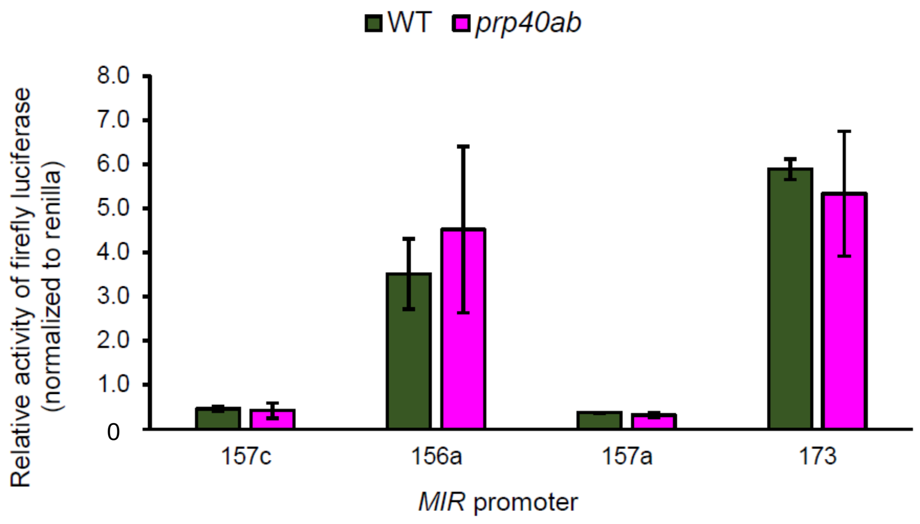

Supplementary Figure S16. *MIR* promoter activity does not depend on AtPRP40. The activity of firefly luciferase produced under the *MIR* promoter was measured in transfected Arabidopsis wild-type (WT) and *prp40ab* protoplasts. To control transfection efficiency differences, the firefly luciferase activity was normalized to *renilla* luciferase produced from the same plasmid. Data represent means ± SD (n = 3). Supports Figure 4.

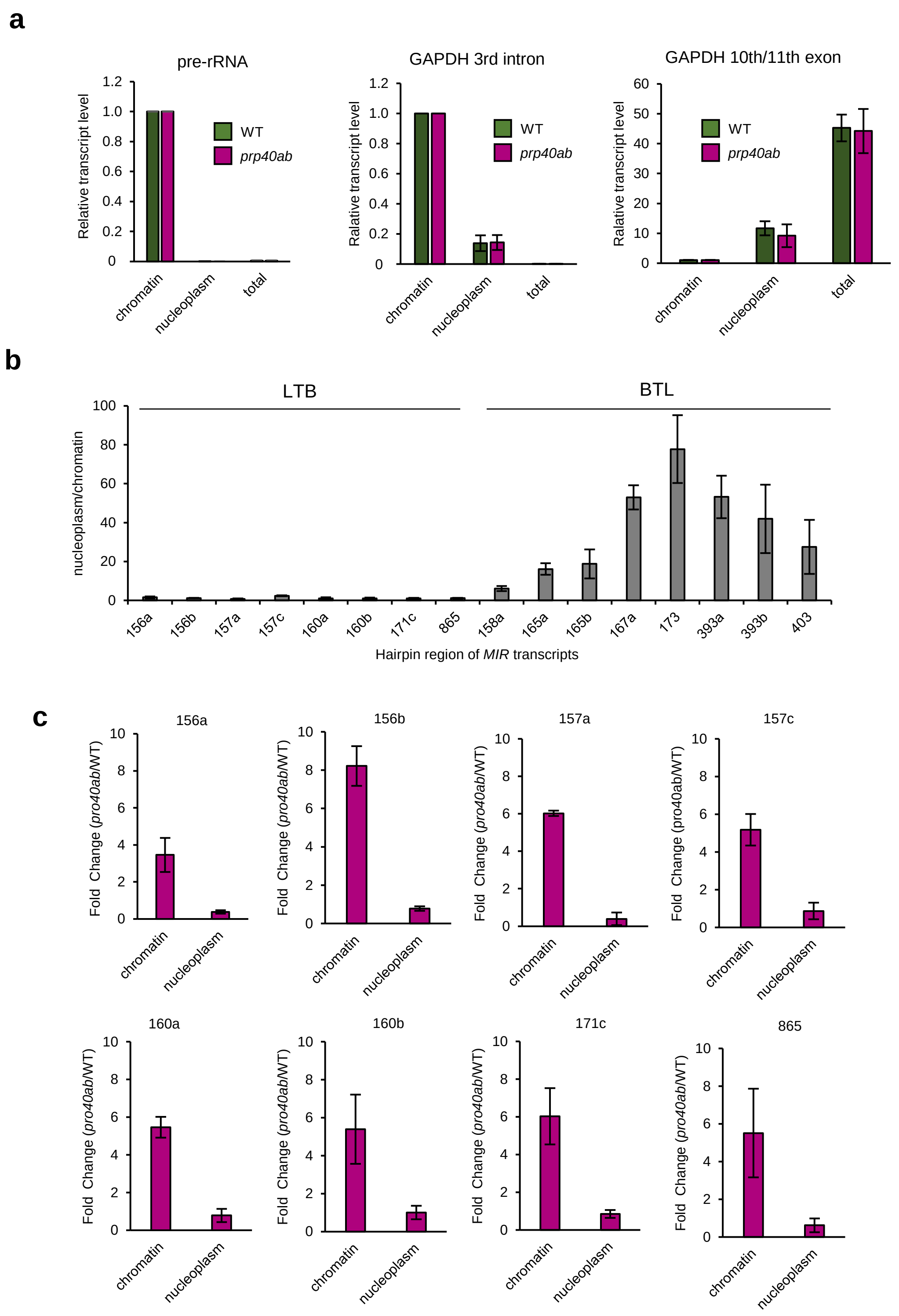

Supplemental Figure S17 Continued on the next page.

d

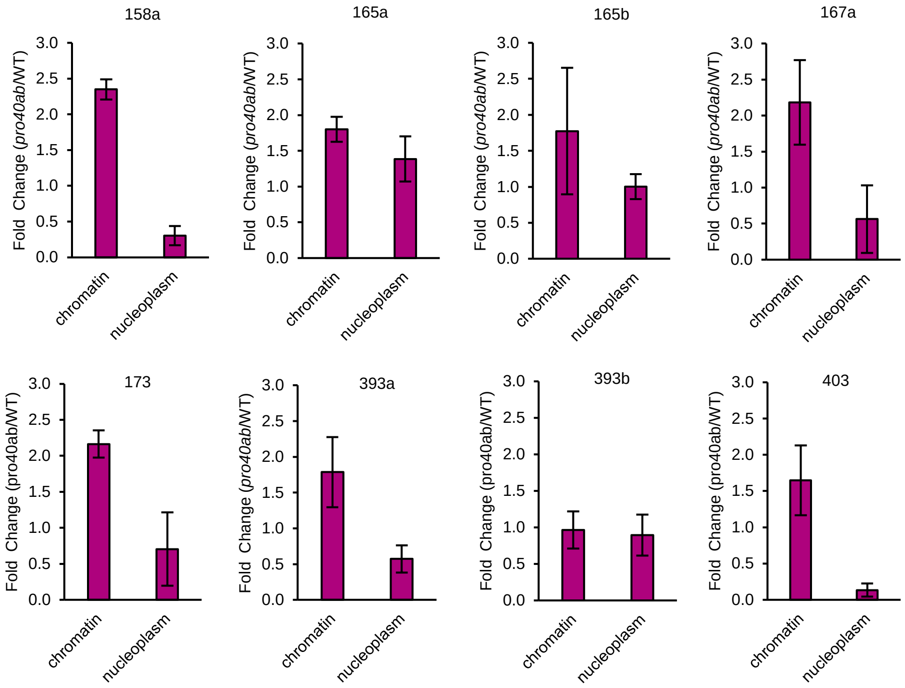

e

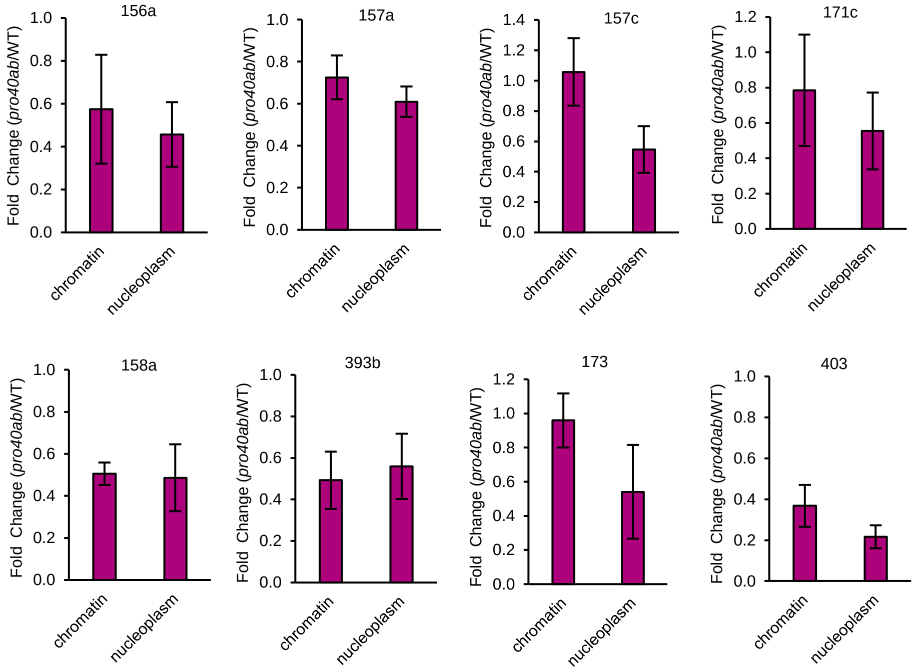

Supplemental Figure S17 Distribution of *MIR* gene transcripts between chromatin and the nucleoplasm is affected in the *prp40ab* mutant. a, Quantitative RT-PCR showing changes in the distribution of control transcripts depending on the tested fractions (n = 3). b, RT-qPCR showing changes in the nucleoplasm/chromatin ratio of the hairpin region depending on the pri-miRNA processing type (LTB, loop-to-base; BTL, base-to-loop) (n = 3) in wild-type (WT) plants. c, Loop-to-base-type miRNA precursor fold change (*prp40ab*/WT) for chromatin and nucleoplasmic fractions (n = 3). Primers amplifying hairpin fragments between miRNA and miRNA* were used. d, Base-to-loop-type miRNA precursors fold change (*prp40ab*/WT) for chromatin and nucleoplasmic fractions (n = 3). Primers amplifying hairpin fragments between miRNA and miRNA* were used. e, Polyadenylated pri-miRNA fold change (*prp40ab*/WT) for chromatin and nucleoplasmic fractions (n = 3). Primers amplifying hairpin fragments between miRNA and miRNA* were used. Data represent means ± SD. Supports Figure 5.

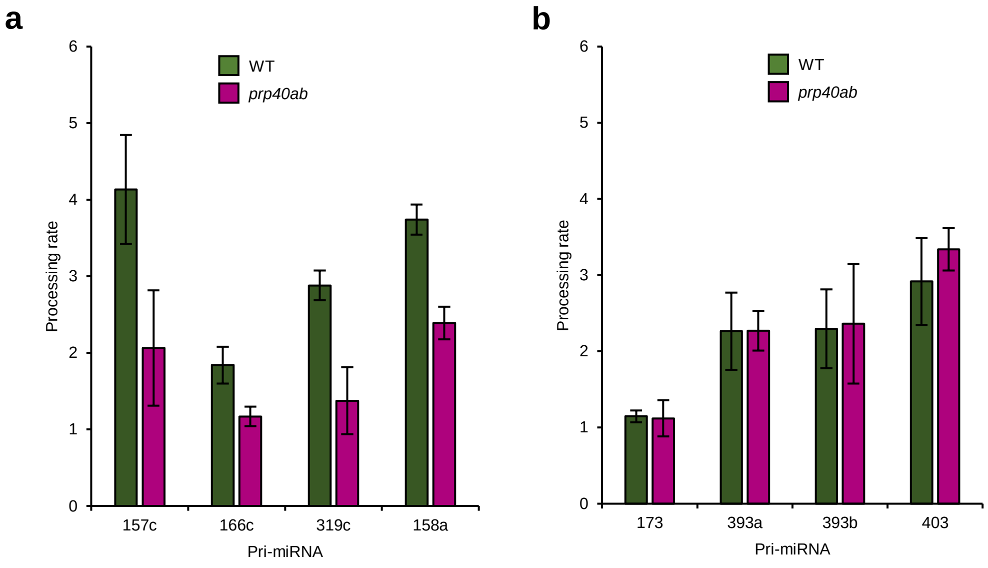

Supplemental Figure S18. Cotranscriptional processing rate in *prp40ab* mutant plants depends on DCL1 distribution on *MIR* genes. Cotranscriptional processing was measured with RT-qPCR as the relative abundance of the loop/hairpin region over the amount of unprocessed pri-miRNAs (primers flanking the DCL1 cleavage site) for a, *MIR*s with increased DCL1 occupancy in *prp40ab* plants and b, without increased DCL1 occupancy in *prp40ab* plants. Data represent means ± SD (n = 3). Supports Figure 5.

Sup plemental Table 1. Primers used in this study

Name	Forward Sequence (5' → 3')	Reverse Sequence (5' → 3')	Localization
GAPDH_i3	TTCGTTTCTTCTTCTCTTCGAT	TGATAAACAGCACACACCATCA	3rd intron
GAPDH_e9	TGGAAAATTGACCGGAATGT	TCGTCGTATGTTGCAGCTTT	9th exon
GAPDH_e10/11	TTGGTGACAACAGGTCAAGCA	AAACTTGTCGCTCAATGCAATC	10th/11th exon
pre_rRNA	GCGAACCAAAGATCACCACT	TTCGTTTGCATGTTCCTTGA	
intergenic	AGTTCAATGGAGAGATGTCGAAATATG	AAGAGGAAAAGAAAGAGATGGAGAGA	
PRP40a	ACACTTGGCCTGTTCCTGTT	TGCGGAGTCAAGTTTCCTGG	4th/5th exon
PRP40b	AAGCACCGTGATGAGTTCCA	CCAGAGGAGTTTGACGCAATTG	21st/22nd exon
PRP40c	CAAGGAATGTGGTTGCAGCC	CCAAGCGGATGAGAACCAGA	2nd exon
At4g24740	CTCTGAGGCATCCGTTTTTC	GGGGTTGTGGGGTAAAGACT	12th exon
At4g39260	CGAAATGTTAATTCGCGTGT	TCCTTTAGCTGCTGTTCGATT	4th exon
At1g10020	AAGGGCGCTAACAAAATAGGA	TCGTAGAACCCAAACCCAAC	2nd exon
At5g07220	AGGCACTCGACCTCTTGAAA	TAGGCTCCTCCTCCTCTTCC	4th exon
At3g19540	CAGGGCTCTTGGTTCACATT	ATGATTCCTTCGACCTGACG	3rd exon
At5g19120	GTGGAGGATTCATGGGAAGA	CAGAGCGTGTTCTTTGTCCA	
At5g61590	TGCAAATGGAAGCAGAATCA	CACGCCTCTGTAATGTCTCG	
At1g80440	TGTCTCCTTCGCTCCTCCTA	CAGGCGTAGCGATGATCTTT	
At1g14920	CGACCGAAGCCAAACTAAAT	CCTCCAATGTCACATGCAAA	
AT4G39400	GTTTGACCCCGAGCTTATGA	GACCCGGCTTGTATCTCCTT	
MIR 156a	GAAGAGAGTGAGCACACAAAGGC	CAGAAAGAGCAGTGAGCACGC	hairpin
MIR 156b	GACAGAAGAGAGTGAGCAC	GACAGAAAGAGAGGTGAGCACGCAC	hairpin
MIR 157a	GCCTTATGCCATTGAAAAACTGG	GCAAAGAACATGCTCCGAATTG	pri-miRNA
MIR 157a	CAGAAGATAGAGAGCACAGATG	GACAGAAGGCTAGAGAGCAC	hairpin
MIR 157c	TGCTCTCTATACTTCTGTCACCACCTT	GCACCCATGTTAGTATTACGCATAGAT	pri-miRNA
MIR 157c	GAGCACTAAGGATGACATGCAA	GCAGGTTCTCTCTCTTTCTCTCC	hairpin
MIR 157c	ACACCGCATGTGGATGATAA	TAGAGATGCAGGTTCTCTCTCTTTC	loop
MIR 157c	GGTTTGAGAGTGATGTTGGTTG	TAGAGATGCAGGTTCTCTCTCTTTC	DCL1 cleavage site
MIR 158a	TGAACAAGGGCATCTAAAGTCACA	TTACTGGACCACGAATTCACCAT	pri-miRNA
MIR 158a	TTTGTCTACAATTTTGGAAAAAG	GCTTTGTCTACATTTGGGAAAG	hairpin
MIR 158a	GGCTTTACTTTAGATTCTTCTAGGG	GCTTTGTCTACATTTGGGAAAG	DCL1 cleavage site
MIR 159a	TCTTTACAGTTTGCTTATGTCAGATCCA	ACCCTGCTCAACTCATGTTTGAA	pri-miRNA
MIR 159b	TGCTTGGATCTCTAATGCTGTTCA	TCACCCTGCTAAACCCTCCA	pri-miRNA
MIR 160a	ATCGATGACCTCCGTGGAT	ACCTCTAATCTCTTCTACACATGATGA	pri-miRNA
MIR 160a	CTGGCTCCCTGTATGCCATATG	CATGGCTCCTCATACGCCATC	hairpin
MIR 160b	CCTGGCTCCCTGTATGCCACAAGA	GCTTGACTACTCTGTACGCCAC	hairpin
MIR 161	TGACCAGTTTATTGCGTCGATCA	TGCTTTTCCCTCTTTTTACAAATGC	pri-miRNA
MIR 163	AGTACCTTAGATAAACCGACCAAAACC	AACCGGGAACTCCAGCACTT	pri-miRNA
MIR 165a	CCATCATCACCATTCACCAA	CCAGACAACATTCCCCTCAA	pri-miRNA
MIR 165a	GTTGTCTGGATCGAGGATATTATAG	GTCCGAGGATACTCTCTATGATC	hairpin
MIR 165b	TTCTGTTGTGGGGAATGTTG	CAGAGTGATTGAAGGCAATTAACA	pri-miRNA
MIR 165b	GGAATGTTGTTTGGATCGAGG	CCGACGATACCATGTGGCATG	hairpin
MIR 166c	TCATGAAGAAGAGAATCACTCG	GGGTTTTCTTAATTTGTTCTTCCA	loop
MIR 166c	TGCGATTTAGTGTTGAGAGGATTG	GGGTTTTCTTAATTTGTTCTTCCA	DCL1 cleavage site
MIR 167a	TGATCTGCTACGGTGAAGTCTATGG	GAAACTGCGAACATGATCTAATCGA	pri-miRNA
MIR 167a	GCTGCCAGCATGATCTAATTAG	CTGCGAACATGATCTAATCGAG	hairpin
MIR 168a	ATTCGCTTGGTGCAGGTC	TCCGATTCAGTTGATGCAAG	pri-miRNA
MIR 168b	CGCTTGGTGCAGGTCGGGAA	ACGGGAGCTCACAAACCAACCA	pri-miRNA
MIR 171c	ATGTGGATGGAGTTTGGTGTAA	GTGATATTGGCACGGCTCA	pri-miRNA
MIR 171c	GATATTGGTGCGGTTCAATC	GGCACGGCTCAATCAAATAAAC	hairpin
MIR 173	GGTGATTAAGTACTTTCGCTTGC	GCAAGCTCTTTCGCTTACACA	pri-miRNA
MIR 173	GCTTGCAGAGAGAAATCACAGTGG	CTTACACAGAGAATCACAGAGG	hairpin
MIR 173	GGTGATTAAGTACTTTCGCTTGC	CTTACACAGAGAATCACAGAGG	DCL1 cleavage site
MIR 319b	AGAGCTTTCTTCGGTCCACTCAT	CAATTTGTCTCTCGCATCATTCAT	pri-miRNA
MIR 319c	TCATGGATAGAAAAAGAAGAGGGTAGAA	AAAAATATCTCCCGCATCATTCAC	loop
MIR 319c	GATTCTTTCAGTCCAGTCATGG	AAAAATATCTCCCGCATCATTCAC	DCL1 cleavage site
MIR 393a	GCATTGATCCTAATTAAGGTG	TAGCATGATCCAAAACCAAGC	hairpin
MIR 393a	GACAAAAACCACATTGCTCTCA	TAGCATGATCCAAAACCAAGC	DCL1 cleavage site
MIR 393b	TCGCATTGATCCTAATTAAGCTG	CGCATGATCCGGAAAAGTAA	hairpin
MIR 393b	ATTGCTCCCACCTTGAAAGA	CGCATGATCCGGAAAAGTAA	DCL1 cleavage site
MIR 396a	ACATGACCCTCTCTGTATTCTTCCA	CAATCGAGCAGAGATATGAAGAAAATC	pri-miRNA
MIR 396b	CCACAGCTTTCTTGAACTTTCTTTTTC	TTGTCATGTTTTCCCACAGCTTT	pri-miRNA
MIR 398a	GCAGTGAAAGCAAAGTATGGAG	TGGAACAGGGGAGATTCAAAGGG	pri-miRNA
MIR 399a	CATTGTACCGTGGAATATGCTTCTT	CCTGCCAATAGAGATCTTACCCTGTA	pri-miRNA
MIR 403	AGAGTCGTATTACATGTTTTGTGCTTGA	ACAGATTACGAGTTTGTGCGTGAA	pri-miRNA
MIR 403	GTTTTGTGCTTGAATCTAATTCAAC	GTTTGTGCGTGAATCTAATCCAAC	hairpin
MIR 403	GACGATTGTCATTAGAAGAGTCG	GTTTGTGCGTGAATCTAATCCAAC	DCL1 cleavage site
MIR 779	GCTGAGCTTTCTCGTCATCACTTATT	GGTGATACAACATAATGAGCAGCAA	pri-miRNA
MIR 865	GGATCTAATTGAGCAAAAAATTG	GAGGAAAAATATGCGAAATTC	hairpin
MIR 156a	CGCGGATCCATGTCAATGTATCTAGAGATAATATTC	ACGCGTCGACGTTTCTTTGCGTTTCTCTTGTCCCAAC	promoter
MIR 157a	CGCGGATCCATGAAGATAGGGTTGAGAAAGC	ACGCGTCGACCACTATCAATGCCTCTCAATTCTC	promoter
MIR 157c	CGCGGATCCGAAACGCACCAACCATTCTT	ACGCGTCGACAAAGGGAAAATAGAGAGTAATGTGTA	promoter
MIR 173	CGCGGATCCTCACAATTCGATCGTCATTACA	ACGCGTCGACTTGTGAGAAGAAGATCAGTAGAAGAAGA	promoter

For pri-/hairpin amplification mirEX platform primers were used (Zielezinski, A. *et al.* 2015)

Supplemental Data. Stepien et al. (2022). Plant Cell.

Figure	Statistical test used	p-value
Figure 4 b	Mann–Whitney U test	< 0.0001
Figure 4 d	Mann–Whitney U test	0.040

Figure	Statistical test used	p-value
Figure 5 a	Mann–Whitney U test	0.004
Figure 5 b	Mann–Whitney U test	0.006
Figure 5 c	Mann–Whitney U test	0.216
Figure 5 d	Mann–Whitney U test	0.000

Supplemental Figure S7 — Student’s t test

SE + RNAPII in *prp40ab*	p-value			
	**PEARSON**	**LI Q**	**SPEARMAN**	**Colormap**
Ser5	1.36E-45	4.48E-15	1.86E-43	7.99E-17
Ser2	5.53E-25	3.44E-10	1.62E-08	1.30E-10

Supplemental Figure S8 — Student’s t test

AtPRP40 + RNAPII in *se-2*	p-value			
	**PEARSON**	**LI Q**	**SPEARMAN**	**Colormap**
Ser5	0.279	0.168	0.317	0.416
Ser2	0.244	0.104	0.479	0.393

Supplementary Figure S12 — Student’s t test

pri-miRNA		156a	158a	159b	160a	160b	161	165a	165b	168a	171c	393a	393b	396a	403
p-value	*prp40ab*	0.004	0.002	0.015	0.025	0.005	0.002	0.001	0.022	0.007	0.016	0.001	0.003	0.021	0.009
	prp40a	0.650	0.152	0.576	0.484	0.258	0.318	0.577	0.738	0.093	0.152	0.198	0.159	0.168	0.053
	prp40b	0.141	0.429	0.294	0.767	0.225	0.425	0.104	0.058	0.082	0.205	0.840	0.132	0.140	0.743
	prp40c	0.687	0.875	0.134	0.956	0.064	0.521	0.662	0.391	0.129	0.354	0.263	0.104	0.338	0.524

Supplementary Figure S16 — Student’s t test

MIR promoter activity in *prp40ab*	p-value
157c	0.701
156a	0.541
157a	0.216
173	0.718
